# Identification and Validation of a GPX4-Related Immune Prognostic Signature for Lung Adenocarcinoma

**DOI:** 10.1155/2022/9054983

**Published:** 2022-05-17

**Authors:** Zhenxing Feng, Bo Li, Qingliang Chen, Hong Zhang, Zhigang Guo, Jianwen Qin

**Affiliations:** ^1^Department of Radiology, Tianjin Chest Hospital, Tianjin 300222, China; ^2^Department of Cardiovascular Surgery, Tianjin Chest Hospital, Tianjin Cardiovascular Disease Research Institute, Tianjin 300222, China; ^3^Respiratory and Critical Care Medicine, Tianjin Chest Hospital, Tianjin 300222, China

## Abstract

Lung adenocarcinoma (LUAD) is a commonly occurring histological subtype of lung cancer. Glutathione peroxidase 4 (GPX4) is an important regulatory factor of ferroptosis and is involved in the development of many cancers, but its prognostic significance has not been systematically described in LUAD. In this study, we focused on developing a robust GPX4-related prognostic signature (GPS) for LUAD. Data for the training cohort was extracted from The Cancer Genome Atlas, and that for the validation cohort was sourced from the GSE72094 dataset including 863 LUAD patients. GPX4-related genes were screened out by weighted gene coexpression network analysis and Spearman's correlation analysis. Then, Cox regression and least absolute shrinkage and selection operator regression analyses were employed to construct a GPS. The ESTIMATE algorithm, single-sample gene set enrichment analysis (ssGSEA), and GSEA were utilized to evaluate the relationship between GPS and the tumor microenvironment (TME). We constructed and validated a GPS premised on four GPX4-related genes (KIF14, LATS2, PRKCE, and TM6SF1), which could classify LUAD patients into low- and high-score cohorts. The high-risk cohort presented noticeably poorer overall survival (OS) as opposed to the low-risk cohort, meaning that the GPS may be utilized as an independent predictor of the OS of LUAD. The GPS was also adversely correlated with multiple tumor-infiltrating immune cells and immune-related processes and pathways in TME. Furthermore, greater sensitivity to erlotinib and lapatinib were identified in the low-risk cohort based on the GDSC database. Our findings suggest that the GPS can effectively forecast the prognosis of LUAD patients and may possibly regulate the TME of LUAD.

## 1. Introduction

Lung cancer is the main contributor to deaths from cancer and is the most commonly diagnosed malignancy around the globe [1]. Among its subtypes, lung adenocarcinoma (LUAD) has emerged as the most common subtype over the last 15 years [2]. Recent advances including the use of targeted therapy and immunotherapy, as well as the identification of oncogenes, have transformed the management of LUAD. However, LUAD is still associated with a low survival rate [3]. The outcome of LUAD is variable and difficult to forecast. While innovative strategies for detecting LUAD and stratifying its prognosis are being developed, the novel biomarkers and risk evaluation models still lack prognostic capability, thereby curtailing the scope for individualized treatment.

Ferroptosis is dissimilar from other kinds of cell death in genetic, biochemical, and morphological terms. It plays a unique role in several cancer biological processes, including autophagy, metabolism, and immune functions in cancer cells [4, 5]. As a selenoenzyme, glutathione peroxidase 4 (GPX4) reduces membrane phospholipid hydroperoxides in order to sustain cellular redox homeostasis, with its cofactor being glutathione [6]. GPX4 is an important regulator of ferroptosis and functions as a carcinogen by impeding ferroptosis in tumor cells [7, 8]. GPX4 was found to be upregulated in several tumor tissues and inversely associated with patient survival based on pan-cancer analysis using The Cancer Genome Atlas (TCGA) [9, 10]. The triggering of ferroptosis by the inhibition of GPX4 has been recognized as a treatment approach to initiate cancer cell death [11]. Recently, GPX4 was discovered to be associated with resistance to anticancer drugs such as cisplatin, as well as “EGFR tyrosine kinase inhibitors (EGFR-TKIs), in non-small-cell lung cancer (NSCLC)” [9, 12, 13]. More critically, increasing evidence has illustrated that GPX4 is associated with the regulation of tumor immune responses [5]. As a metabolic checkpoint, GPX4 in cancers was found to protect activated CD8^+^ T cells and Treg cells from uncontrolled ferroptosis without compromising their function [14, 15]. Besides, GPX4 influences the innate immune system by regulating natural killer and dendritic cells in p53-mutant NSCLC [16]. However, very few studies have systematically evaluated GPX4-related ferroptosis models to forecast the overall survival (OS) in patients with LUAD.

In the present study, we began by identifying the differential GPX4-related genes by weighted gene coexpression network analysis (WGCNA) and Spearman's correlation, using TCGA data on the mRNA expression of LUAD. We then developed and validated a GPX4-related prognostic signature (GPS) for patients with LUAD, based on the TCGA and GSE72094 datasets. Additionally, we assessed the correlation between the GPS and immune infiltrating cells in the tumor microenvironment (TME) of LUAD. Finally, the function of GPS in the response of LUAD to targeted therapy was also evaluated.

## 2. Methods and Materials

**Figure 1:**
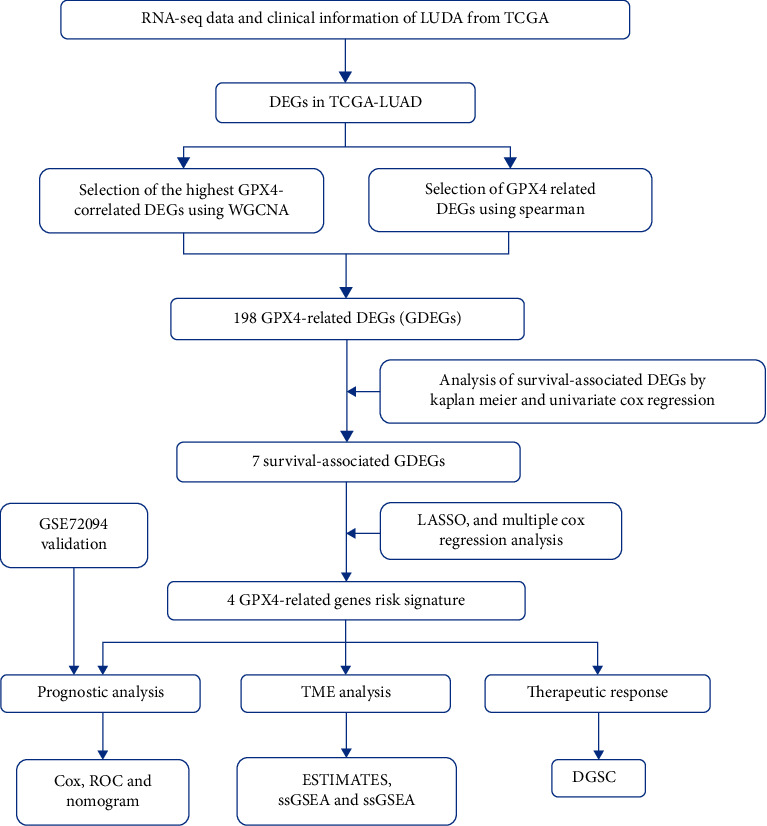
Study design and workflow.

The workflow of this study is shown in Figure 1.

### 2.1. Datasets from TCGA and Gene Expression Omnibus

Profiles on the LUAD gene expression were extracted from the TCGA and Gene Expression Omnibus (GEO) databases. All LUAD data including the associated clinical data were downloaded freely from the TCGA. Out of a total of 594 LUAD samples, 535 were those of LUAD, and 59 were those of normal tissue. For each lung cancer case, transcriptome profiling (RNA-Seq, HTSeq-FPKM) files were downloaded from TCGA. Additionally, the GSE72094 normalized expression profiles, another LUAD gene expression profile, was extricated from GEO [17]. The GSE72094 dataset consisted of 442 patients with LUAD and included their clinical information, EGFR Sanger sequencing data, and detailed mRNA expression data, which were studied on the GPL15048 platform. LUAD patients with complete survival data and a survival time of over 30 days were included in the subsequent analyses. The clinical features of patients with LUAD in the TCGA and GSE72094 datasets examined in this study are summarized in Table S1.

### 2.2. Identification of Differentially Expressed Genes (DEGs) between LUAD Tissues and Normal Tissues

To identify the DEGs between LUAD and normal tissues, the Wilcoxon test method using R package “limma” (version: 3.6.3, The R Foundation for Statistical Computing, Vienna, Austria) was employed to screen out DEGs in the TCGA-LUAD database. The established thresholds were |log2‐fold change (FC)| > 1.0 and false discovery rate (FDR) < 0.05. The DEGs of the TCGA-LUAD dataset were visually represented as heat map and volcano plots using the R package “ggplot2.”

### 2.3. WGCNA

WGCNA was employed to examine the gene composition of GPX4-related modules in the samples. Modules with an elevated correlation coefficient were regarded as candidate modules related to GPX4 and were chosen for the ensuing analysis. The “WGCNA” package in R was utilized to construct TCGA-LUAD gene expression profiles to gene coexpression networks [18]. A comprehensive explanation of the WGCNA method has been provided in past reports [19, 20].

### 2.4. Constructing and Validating a GPX4-Related Risk Signature

We began by performing Kaplan-Meier (K–M) and univariate Cox regression analyses for estimation of survival related to GPX4-related DEGs (GDEGs), with *P* < 0.05 in the two algorithms considered to be candidate genes for building the model [21, 22]. Subsequently, least absolute shrinkage and selection operator (LASSO) regression was performed to ulteriorly reduce the number of gene candidates. Ultimately, we developed a GPX4-related risk scoring system and multiplied the normalized level of expression demonstrated by each highly GDEG, with the regression coefficients obtained from the multivariate Cox analysis. The high-risk and low-risk cohorts were classified using the TCGA-LUAD median risk score. K–M survival analysis, along with the time-related receiver operating characteristic (ROC) curve analysis, was employed to assess the prognostic ability of the model. Multivariate and univariate Cox regressions were executed to ascertain if the risk score could forecast the prognosis independently. A nomogram was developed and validated for precise forecasting of OS using “survival” and “regplot” packages in R. The TCGA-LUAD dataset was used as the training cohort, whereas the GSE72094 was utilized as the validation cohort.

### 2.5. Estimation of the Stromal, Immune, and ESTIMATE Scores

Immune score (the proportion of immune components), stromal score (the proportion of stromal components), and ESTIMATE score (the aggregate of the above scores) were computed for the specific LUAD sample utilizing the “ESTIMATE” package in R [23]. A higher score illustrates the substantial quantity of the corresponding component (stromal, immune, or tumor purity) in the TME [24].

### 2.6. Single-Sample Gene Set Enrichment Analysis

Based on single-sample gene set enrichment analysis (ssGSEA) in the TCGA-LUAD cohort, the infiltration scores of 16 immune cells and 13 immune-related pathways were estimated using the software packages “GSVA,” “limma,” and “GSEABase” in R [25, 26].

### 2.7. Gene Set Enrichment Analysis (GSEA)

In order to ascertain the immunological pathways that are considerably altered in LUAD, we undertook GSEA between the high- and low-risk cohorts utilizing GSEA (version 4.1.0). The (c2.cp.kegg.v7.4.symbols.gmt) file was chosen to act as the reference gene file. FDR < 0.05 was chosen to be the minimum limit criterion [27].

### 2.8. Prediction of Targeted Therapy Response

The LUAD patients' response to targeted drugs was forecast using the commonly used Genomics of Drug Sensitivity in Cancer (GDSC) pharmacogenomics database. The half-maximal inhibitory concentration (IC50) was approximated using the R package “pRRophetic” [28].

### 2.9. Statistical Analysis

Spearman's correlation was used for correlation tests, with Spearman's correlation coefficient ≥ 0.2 and *P* < 0.0001 being regarded as significant. KM method and log-rank test in the GraphPad Prism 8.0 software were employed for the survival analysis. R package “survivalROC” was employed to chart ROC curves. The Wilcoxon signed-rank test was used to assess the relationship between classified variables and the risk score, and box plots were generated on the GraphPad software.

## 3. Results

### 3.1. Identification of Highly GPX4-Related DEGs

**Figure 2:**
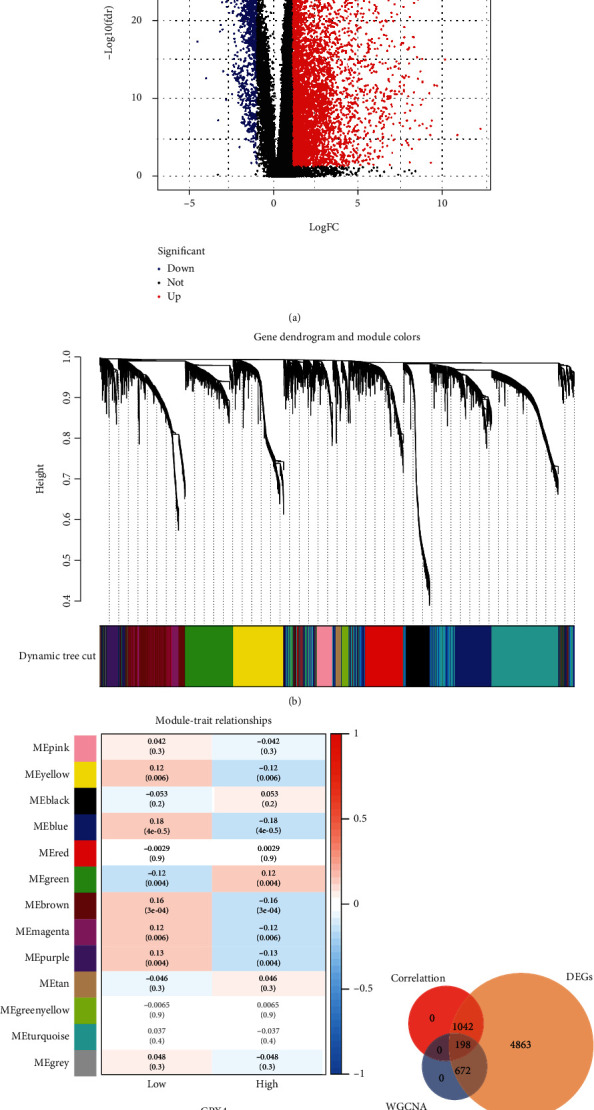
Identification of GPX4-related differentially expressed genes (DEGs) in The Cancer Genome Atlas dataset of lung adenocarcinoma. (a) DEG volcano map. (b) The cluster dendrogram for coexpression network modules. (c) Module-trait relationships. (d) The Venn diagram among DEG lists, coexpressed blue module, and GPX4-related genes.

A total of 6,775 DEGs in the TCGA database (Figure S1, Figure 2(a)) were revealed as being dysregulated in LUAD tissues than in normal tissues. In order to identify the highly GDEGs in patients with LUAD, WGCNA and Spearman's correlation analyses were performed based on the TCGA-LUAD. Each of the modules was allocated a color and an aggregate of 13 modules in the TCGA-LUAD (Figure 2(b)) was discovered in this study. Subsequently, a module-trait relationship heat map was plotted to investigate the correlation between each module and GPX4 expression (either low or high). The outcomes of this module-trait relationship are displayed in Figure 2(c), depicting that the blue module (870 DEGs) in the TCGA-LUAD dataset had the highest association with GPX4 expression (blue module: *r* = 0.18, *P* < 0.0001). In addition, based on Spearman's correlation analysis, a total of 1,240 GPX4-associated DEGs were screened out in the TCGA dataset (Table S2, *P* < 0.001). In total, 198 overlapping genes were extricated as highly GDEGs for subsequent prognostic analysis, presented as a Venn diagram in Figure 2(d).

### 3.2. Construction of GPS in TCGA-LUAD Dataset

Using univariate Cox regression and K–M survival analysis, we recognized seven GDEGs linked to OS of LUAD in the TCGA dataset (Table S3). Also, we employed multi-Cox regression and LASSO regression to reduce model genes' scope and optimize the model (Figure S2). Lastly, a GPS comprising of four highly GDEGs was constructed (Table S4). The risk score was calculated as follows:

Risk score = (0.1038 × expression_KIF14_) + (0.0577 × expression_LATS2_)–(0.2683 × expression_PRKCE_)–(0.2043 × expression_TM6SF1_).

**Figure 3:**
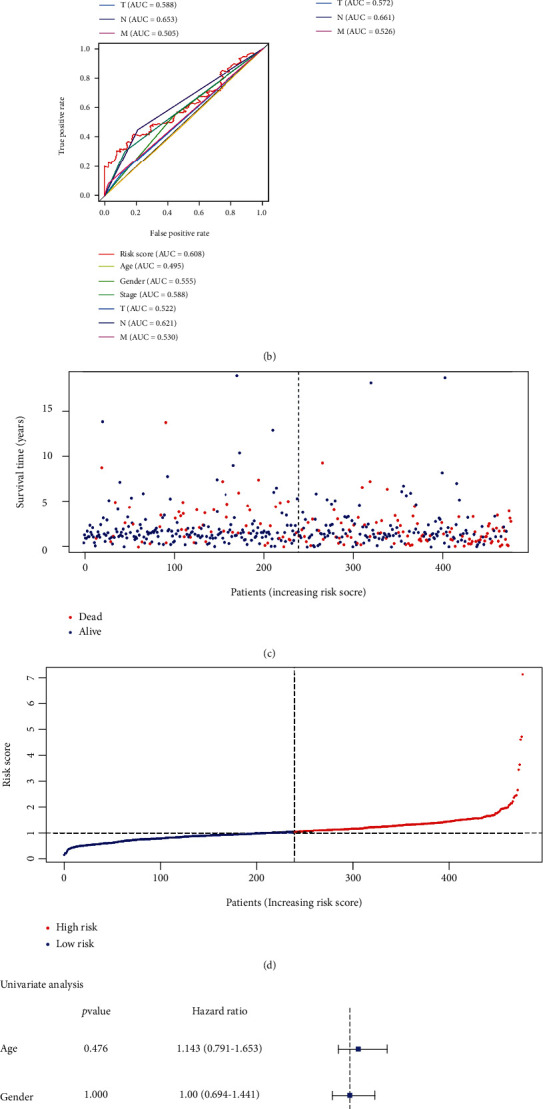
Construction of the GPX4-related prognostic signature based on the TCGA-LUAD dataset. (a) The analysis of patients' overall survival (OS). (b) The time-dependent receiver operating characteristic analysis of the risk score. (c, d) The distribution of risk score and the survival status of patients. (e, f) Forest plot for univariate and multivariate Cox analyses of OS in lung adenocarcinoma.

The median risk score of the TCGA-LUAD training cohort was used as the integrated cut-off for separating the low-risk cohort from the high-risk cohort. The high-risk cohort exhibited a considerably worse OS compared with the low-risk cohort (Figure 3(a)), and the area under the curve (AUC) of the GPS at, 1, 3, and 5 years was 0.759, 0.682, and 0.608, respectively (Figure 3(b)). The mortality risk in LUAD patients exhibited a rise with the increase in the risk model score (Figures 3(c) and 3(d)). The results of the univariate and multivariate Cox analyses illustrated that the GPS could independently forecast the OS in the TCGA-LUAD dataset (Figures 3(e) and 3(f)).

### 3.3. Validation of GPS in the GSE72094 Dataset

**Figure 4:**
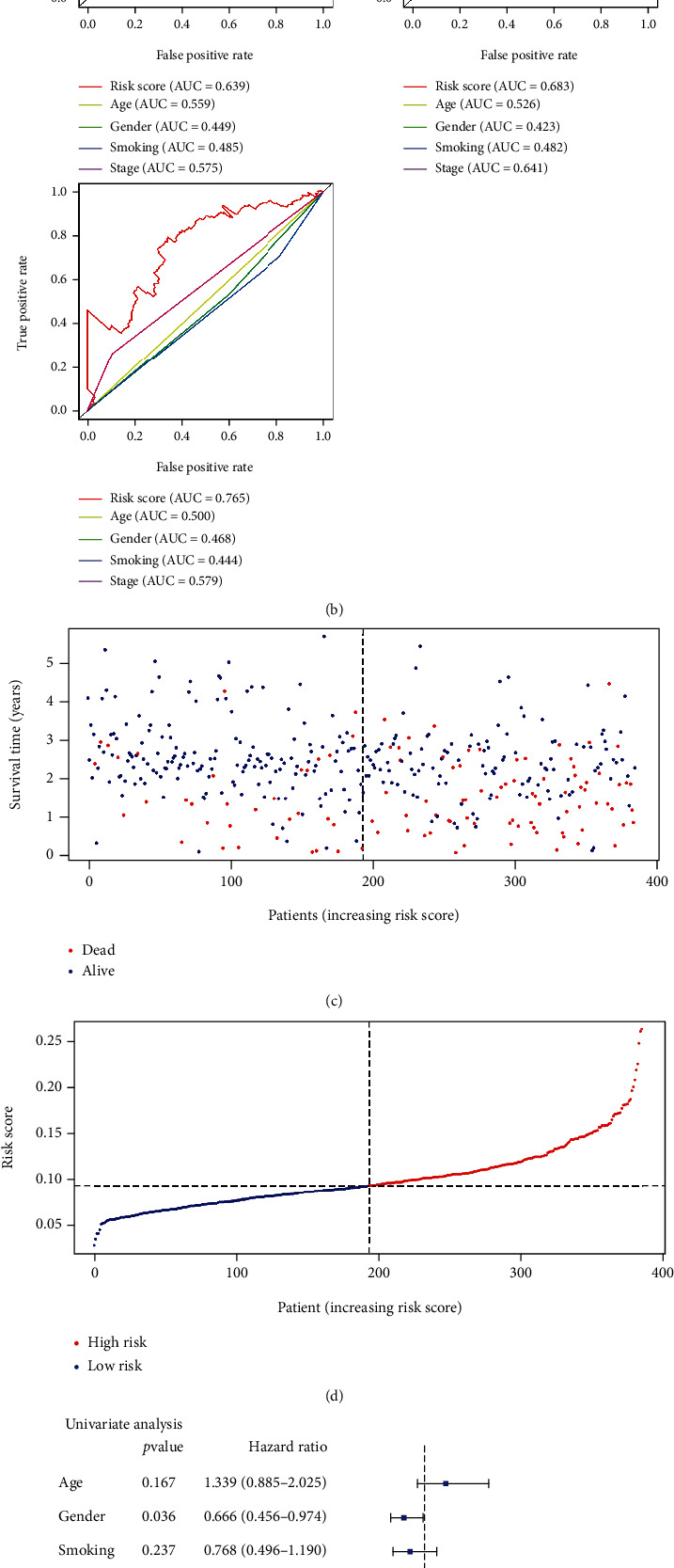
Validation of the GPX4-related prognostic signature in the GSE72094 dataset. (a) The Kaplan-Meier curve of overall survival in the GSE72094 dataset. (b) Time-dependent receiver operating characteristic analysis for risk score in the GSE72094 dataset. (c, d) Risk score distribution and patient survival status in the GSE72094 dataset. (e, f) Forest plot of the multivariate and univariate Cox analyses of overall survival in lung adenocarcinoma in the GSE72094 dataset.

For verifying the robustness of our GPS, we performed external validation on a large independent cohort of patients with LUAD in the GSE72094 dataset (*n* = 386). In line with the outcomes of TCGA, patients with high-risk scores displayed considerably poorer OS compared to those with low-risk scores (Figures 4(a), 4(c), and 4(d)). In the GSE72094 dataset, the AUC at 1, 3, and 5 years was 0.639, 0.683, and 0.765, respectively (Figure 4(b)). The multivariate and univariate Cox regression analyses demonstrated that the GPS risk score could independently predict the OS in the GSE72094 dataset (Figures 4(e) and 4(f)). These results further suggest that the GPS we developed was capable of general application.

### 3.4. Association between GPS and Clinicopathological Features

**Figure 5:**
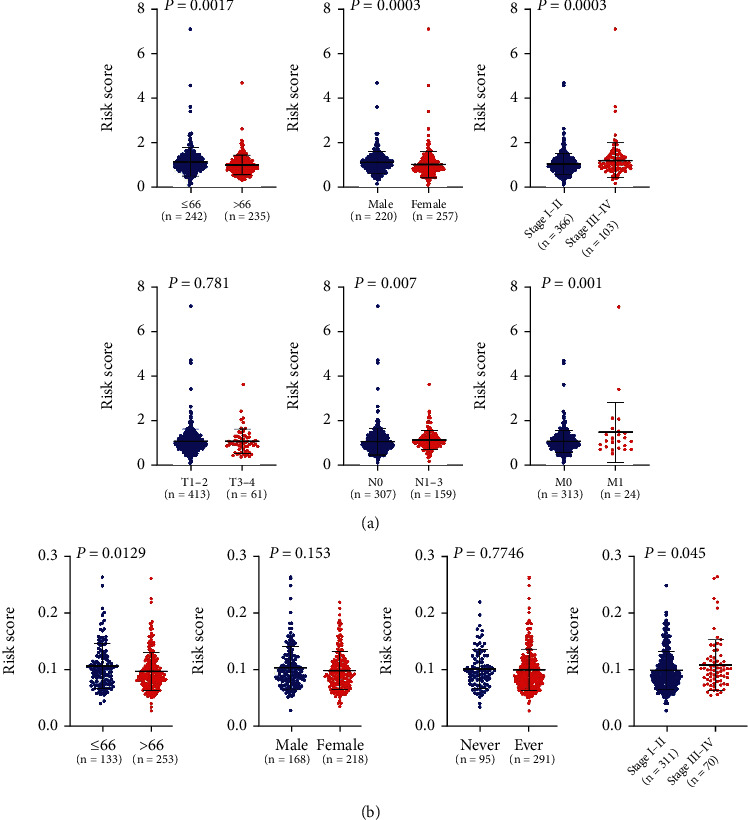
The relation between GPX4-related prognostic signature and clinical features in lung adenocarcinoma. (a) Sex, age, M stage, N stage, T stage, and American Joint Committee on Cancer (AJCC) stage in the TCGA-LUAD dataset. (b) Age, sex, smoking status, and AJCC stage in the GSE72094 dataset.

**Figure 6:**
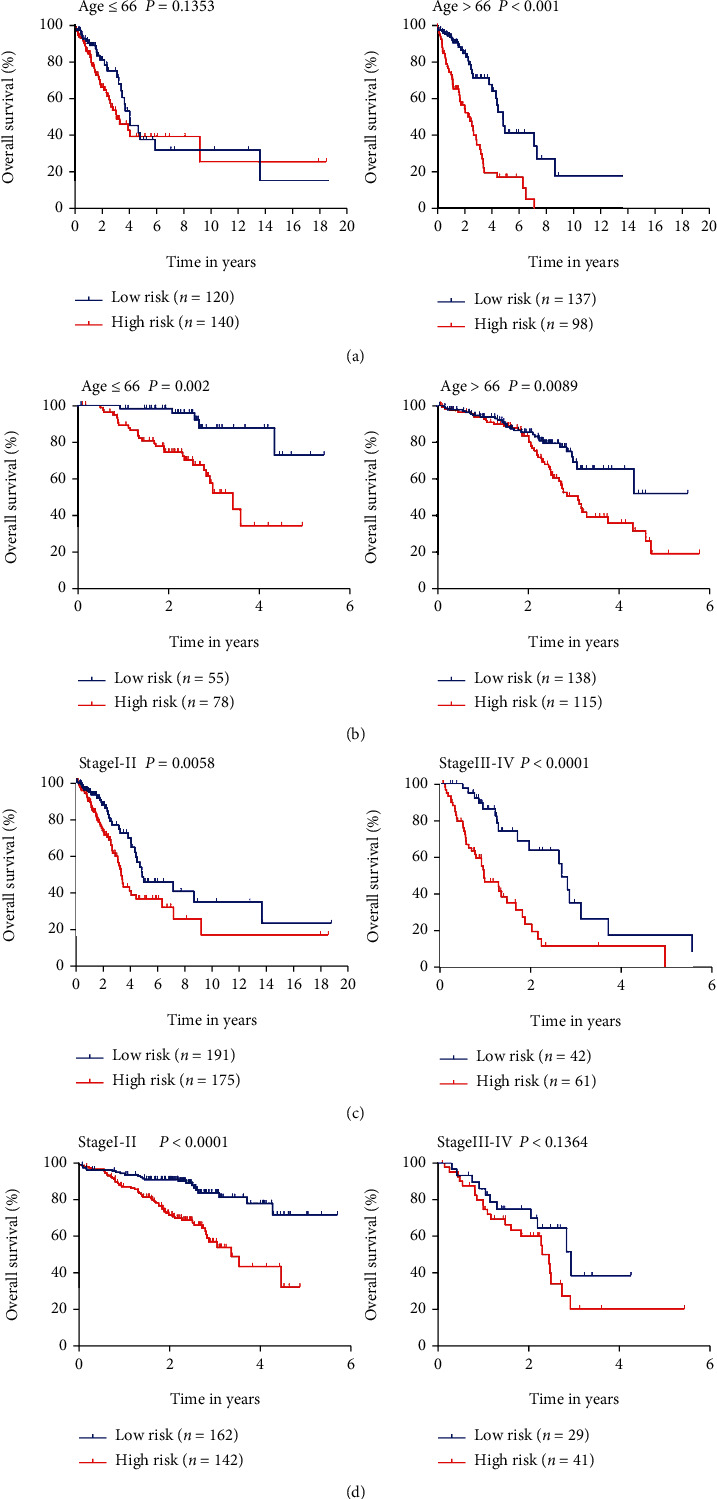
K–M survival subgroup analysis of all patients with lung adenocarcinoma based on the GPX4-related prognostic signature stratified by TNM stage. (a) Age in the TCGA-LUAD dataset. (b) Age in the GSE72094 dataset. (c) Stage in the TCGA-LUAD dataset. (d) Stage in the GSE72094 dataset.

This study assessed the relationship between GPS and the prognostic factors using the clinical data of two separate datasets. In the TCGA-LUAD dataset, elevated risk scores were significantly correlated with age, sex, advanced TNM stage, N stage, and M stage (tumor metastasis) (Figure 5(a)). Similarly, in the GSE72094 dataset, the two groups were considerably distinct with respect to the age and TNM stage based on Figure 5(b). The above results indicated that age and TNM stage can effectually forecast survival in patients with LUAD. Therefore, to study the GPS prognostic value stratified by age and TNM stage in these patients, subgroup analysis was performed. As illustrated by the K–M curves, GPS remained a stable prognostic factor in patients with LUAD who were graded by age (Figures 6(a) and 6(b)) and TNM stage (Figures 6(c) and 6(d)), though larger groups are required for further validation.

### 3.5. Establishment and Validation of a Nomogram

**Figure 7:**
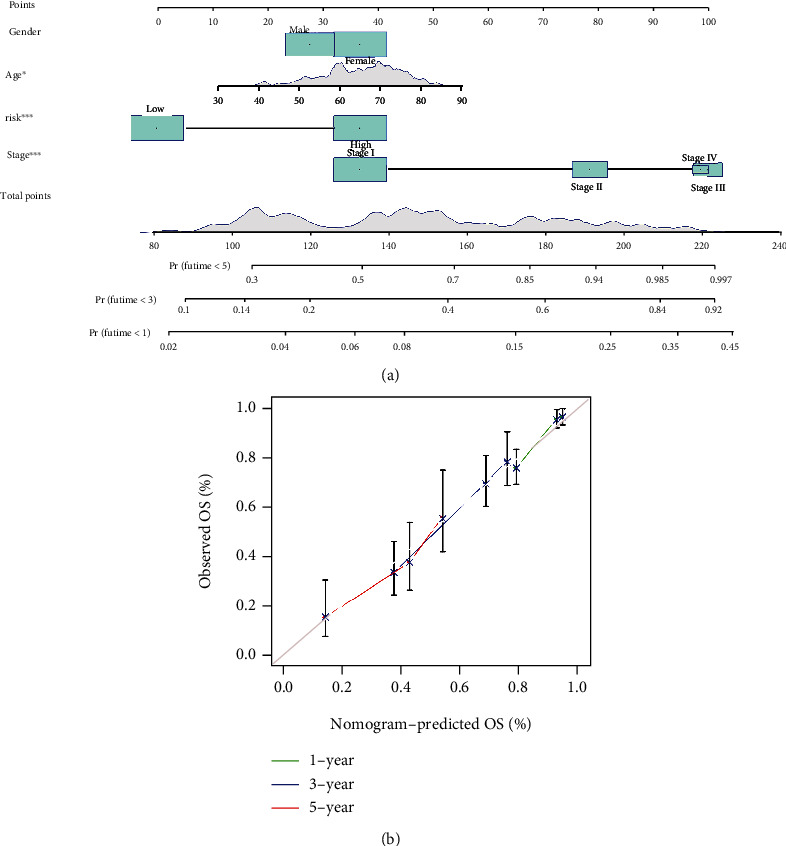
The nomogram for forecasting the overall survival probability of patients with lung adenocarcinoma. (a) The nomogram plot. (b) The nomogram calibration curves in the years 1, 3, and 5.

In order to come up with a quantitative method that could forecast the OS of LUAD patients, two nomograms, including a number of clinicopathological features (age, sex, risk score, and pathological stage) were built based on the TCGA-LUAD dataset (Figure 7(a)) and GSE72094 dataset (Figure S3A). We also assessed the predictive performance of GPS with the above clinical features using time-dependent ROC curves (Figures 3(b) and 4(b)) and a calibration plot (Figure 7(b) and Figure S3B). The results of the ROC and calibration plots showed that GPS had better predictive power and accuracy compared with other clinical attributes such as sex, age, TNM stage, and grade.

### 3.6. Relationship between GPS and Tumor-Infiltrating Immune Cells in TME

**Figure 8:**
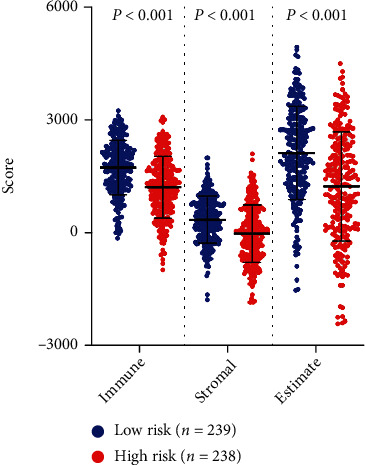
Comparison of ESTIMATE scores in the high-risk and low-risk cohorts.

**Figure 9:**
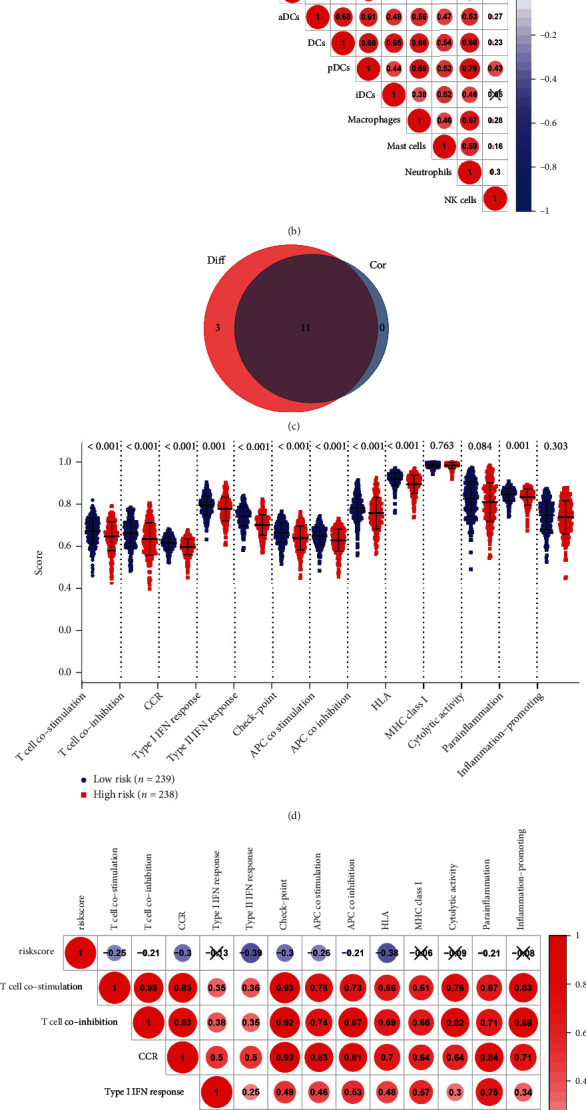
The results of ssGSEA. (a) The scatter dot plot shows the score of 16 kinds of immune cells in the low-risk and high-risk cohorts. (b) Heat map shows the association between risk scores and the 16 types of immune cells. (c) The Venn plot displays 11 kinds of immune cells correlated with the risk score codetermined by correlation and difference tests. (d) The scatter dot plot shows the score of 13 types of immune-related processes between the high-risk and low-risk cohorts. (e) Heat map denotes the correlation between the risk score and 13 kinds of immune-related processes. (f) The Venn plot displays nine kinds of immune-related processes linked to the risk score codetermined by correlation and difference tests.

To determine if the GPS could aid in characterizing the TME in LUAD, we employed the ESTIMATE algorithm to compare the characteristics of gene expression of immune cells and stromal cells between the high-risk and low-risk cohorts. Contrasted with the high-risk cohort, the low-risk cohort exhibited a significantly higher immune score, stromal score, and ESTIMATE score (Figure 8, all *P* < 0.001). We further assessed the relationship between the GPS and immune cell infiltration using ssGSEA in the TCGA-LUAD dataset. Then, a total of 11 types of immune cells including TIL (tumor-infiltrating lymphocyte), T helper cells, Treg, B cells, aDCs, DCs, pDCs, iDCs, neutrophils, mast cells, and macrophages were identified as having a significantly negative association with the risk score from the difference and correlation analyses (Figures 9(a)–9(c), all *P* < 0.05). As for the numerous enriched immune-related activities, we assessed the link between immune-related processes and the risk score based on ssGSEA. We found ten kinds of immune-related processes that had a significant negative correlation with the risk score. They included T cell costimulation, T cell coinhibition, chemokines and chemokine receptors (CCR), antigen-presenting cells (APC) costimulation, APC coinhibition, type II interferon response, human leukocyte antigen, checkpoint, and parainflammation (Figure 9(d)–9(f), all *P* < 0.05). Based on the outcomes, we could ascertain that our GPS was considerably correlated with the immune cell infiltration in TME.

### 3.7. Relationship between GPS and Immune-Related Pathways

**Figure 10:**
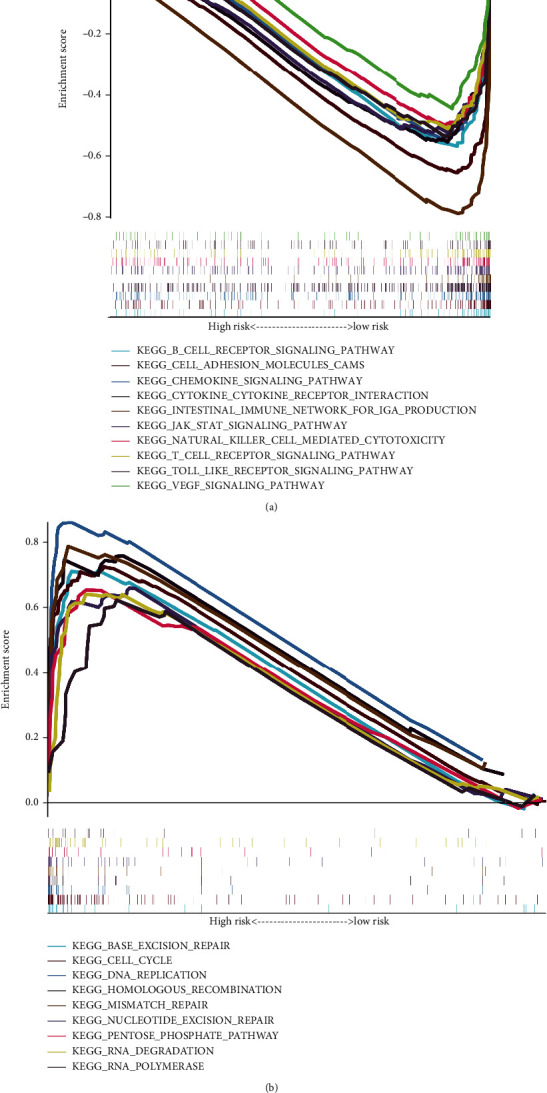
Analysis of gene set enrichment in the low-risk and high-risk cohorts. (a) Gene set enrichment analysis (GSEA) in the group with a low-risk score. (b) GSEA in the group with a high-risk score.

Afterward, GSEA was executed in the high-risk and low-risk cohorts, sequentially, and showed that the gene sets dramatically enriched in the low-risk score cohort were primarily linked to immune-related pathways in LUAD. These included “T cell receptor signaling pathway,” “B cell receptor signaling pathway,” “Natural killer cell-mediated cytotoxicity,” “JAK-STAT signaling pathway,” “Cytokine-cytokine receptor interaction,” and “Chemokine signaling pathway” (FDR < 0.05, Figure 10(a), Table S5). In comparison, none of the gene sets linked to immune pathways was considerably enhanced in the high-risk LUAD cohort. The high-risk cohort was mainly considerably enriched in processes related to tumor repair-associated proliferation in LUAD, including “cell cycle,” “RNA degradation,” “DNA replication,” “base excision repair,” “pentose phosphate pathway,” and “mismatch repair” (FDR < 0.05, Figure 10, Table S6). These findings indicate that the four genes of GPS may be implicated in regulating tumor initiation and progression, as well as immune activity in LUAD.

### 3.8. Relationship between GPS and Targeted Therapy Response

**Figure 11:**
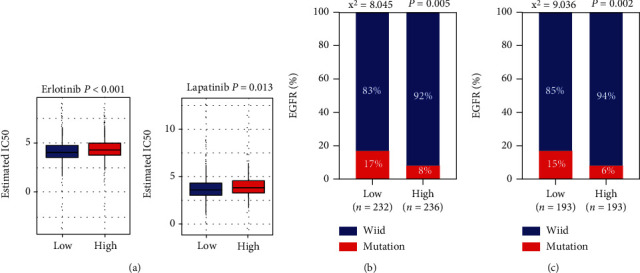
Forecasted responses to EGFR tyrosine kinase inhibitors among different risk cohorts. (a) Comparison of the estimated IC50 values of erlotinib and lapatinib between cohorts with high risk and low risk, respectively. (b) Comparison of the mutation frequency of EGFR between patients with high risk and low risk in the TCGA-LUAD dataset. (c) Comparison of the mutation frequency of EGFR between patients with high risk and low risk in the GSE72094 dataset.

Considering that targeted therapy is an efficacious adjuvant therapy for LUAD, we evaluated the GDSC database to approximate the response of low- and high-risk LUAD patients to molecular-targeted therapy. We found that two commonly used molecularly targeted drugs (erlotinib and lapatinib) had considerable distinctions in the approximated IC50 between cohorts with high risk and low risk. Specifically, patients in the high-risk cohort displayed higher IC50 values for erlotinib, a commonly used EGFR-TKI in LUAD (Figure 11(a), *P* < 0.05). It is known that EGFR mutations in LUAD are related to the efficacy of EGFR-TKIs [29], so we utilized the chi-square test in comparing the mutation frequency of EGFR between the low-risk and high-risk cohorts. As illustrated by the TCGA-LUAD dataset, the mutation frequency of EGFR in low-risk patients was elevated compared to that in the high-risk patients (Figure 11(b), *P* < 0.01). In the GSE72094 dataset, the mutation frequency of EGFR in the low-risk patients was also higher (Figure 11(c), *P* < 0.05).

## 4. Discussion

In the past, studies have used ferroptosis-related risk signatures to classify patients with LUAD into various prognostic subgroups [30–33]. The inventory of ferroptosis-related genes for construction of a prognostic model was usually obtained via differential analyses or from public databases. In contrast, we employed a unique selection approach combining coexpression networks with correlation analysis, which may identify ferroptosis-related genes that had not been previously documented.

As genomics technologies continue to develop, bioinformatics is becoming increasingly popular for studying the molecular processes of disease and for identifying accurate disease biomarkers by analyzing gene expression profiles [34]. A valuable approach for comprehending gene association and gene function from genome-wide expression is WGCNA [18]. WGCNA may be utilized to identify coexpression modules that determine related genes, thus allowing us to forecast the functions of coexpressed genes and identify the genes performing vital functions in cancers [35]. Moreover, Spearman's rank correlation analysis is another influential analysis within transcriptomics that tests the association between two ranked genes in their expression levels [36, 37]. Thus, the results from WGCNA and Spearman's correlation analysis were integrated to improve the recognition of highly correlated genes to GPX4 that are valuable as candidate markers of ferroptosis. Overall, we identified 198 GDEGs for subsequent prognostic analysis.

Then, we employed survival analysis, LASSO regression, and multi-Cox analysis to evaluate GDEGs stepwise and eventually built a GPS with four GPX4-related genes (including KIF14, LATS2, PRKCE, and TM6SF1) based on the TCGA dataset. The GPS' risk score and the risk categorization threshold were considered for categorizing all enrolled patients into high- and low-risk cohorts. Further, K–M survival analysis demonstrated noticeably different prognoses between LUAD patients in the low- and high-risk categories. When patients were graded based on the TNM stage, the GPS still maintained its robustness as a prognostic tool for OS, especially in patients in the early stage. Then, we performed external validation of the GPS by utilizing one independent dataset, GSE72094. Congruent with the TCGA-LUAD outcomes, the OS of the high-risk group was significantly worse than that of the low-risk group. Notably, high risk scores may indicate clinical attributes predictive of lower survival, i.e., advanced distant metastasis, higher TNM stage, and lymph node metastasis, thus providing a rationale for the poor prognosis in patients at high risk. GPS based on multivariate and univariate Cox regression analyses was able to independently predict OS in LUAD in two independent datasets. Following the integration of clinicopathological risk factors and risk cohorts, nomogram was developed and validated for accurate forecasting of OS. The AUC and calibration plot illustrated that our nomogram performed better than TNM staging. These results endorse the general applicability of our GPX4-related prognostic model, thereby verifying its capability in assisting TNM staging for more accurate predictions.

The TME mainly comprises tumor cells and tumor-infiltrating immune cells (TICs) mixed with stromal components. It is turning out to be an imperative part of cancer tumorigenesis and progression and has emerged as a research focus in recent years [24]. Stromal and immune component assessment using ESTIMATE helps predict the clinical outcomes of patients with LUAD, and patients with high immune scores have shown better OS when contrasted with patients with low scores [38]. Interestingly, the ESTIMATE analysis done in our study revealed that GPX4-related risk was negatively correlated with the ESTIMATE score, matrix score, and immune score, suggesting that patients with a low-risk score had abundant immune cell infiltration. TICs have been identified as an important component of TME and have critical consequences for oncogenesis, clinical outcome, and treatment, especially immunotherapy [3, 39, 40]. Moreover, GPX4 has an important function in regulating immune cells in lung cancer and other cancer types [14–16]. Therefore, the immune-related biological characteristics of our GPX4-related risk model were further analyzed using ssGSEA and GSEA. As expected, the risk scores were negatively correlated with the degree of infiltration of 11 types of immune cells (TILs, T helper cells, Treg, B cells, aDCs, DCs, pDCs, iDCs, neutrophils, mast cells, and macrophages) based on ssGSEA. These immunes cells are known to be plentiful in LUAD tissues, where they regulate tumor development, promote the proliferation and invasion of tumor cells, induce metastasis, and also regulate immunotherapy [41–44]. Various immune-related processes have also been found to be more abundant in patients with low-risk scores as per ssGSEA. We deduce that the immunosuppressive TME might be a key factor that contributes to poor prognosis in LUAD patients with high-risk scores. Subsequently, we carried out GSEA to assess the fundamental mechanism of the four-gene GPS in LUAD. GSEA illustrated that the genes of the low-risk group exhibited a high concentration of immune-related biological pathways and processes, as well as tumor repair activities. Collectively, these findings demonstrate that the GPS may be crucial for creation of the immune microenvironment in LUAD.

By analyzing the GDSC database, we discovered that patients with high-risk LUAD showed resistance to erlotinib and lapatinib. Patients diagnosed with EGFR-mutant LUAD are known to exhibit a better initial clinical response to EGFR-TKIs [29]. In addition, we observed that low risk was associated with EGFR mutations in the two independent LUAD datasets. Together, these results show that GPS might be involved in the inefficacy of EGFR-TKIs in LUAD. Interestingly and notably, the results were consistent with those of two recent studies. One study demonstrated that NRF2-GPX4/SOD2 axis conveys resistance to erlotinib in NSCLC cells [12]. Another study confirmed that restrain of GPX4 or mTOR results in loss of lapatinib resistance in NSCLC cells by promoting ferroptosis [13]. All the findings need to be further validated in a laboratory to explore the use of our prognostic model as a predictive marker for the efficacy of EGFR-TKIs treatment in LUAD.

As shown in Table S7, among the four GPX4-related prognostic genes, KIF14 was upregulated, while LATS2, PRKCE, and TM6SF1 were downregulated in the LUAD tissues than in the normal lung tissues. Besides, all of the four genes were adversely associated with GPX4 (Figure S4). The four genes of our signature can act as independent targets, and when combined, may offer improved performance, which is contingent on their prognostic roles and tumor-related characteristics. Among the four genes of the GPS, KIF14, LATS2, and PRKCE have been widely studied in previous studies. For example, kinesin family member 14 (KIF14), a microtubule-dependent cytoskeletal motor protein, is involved in cytokinesis [45]. KIF14 overexpression is linked to several cancers, and KIF14 causes resistance to sorafenib and chemotherapy through the AKT signaling pathway in cancers [46, 47]. KIF14 has been shown to be oncogenic in several studies and has been reported as a prognostic biomarker for several cancers, but its relative importance as a driver gene in lung cancer pathogenesis is yet to be clarified [46–49]. Corson et al. discovered that KIF14 expression was an independent prognostic factor for disease-free survival in NSCLC and knockdown of KIF14 in vitro reduced tumorigenicity [48]. However, in another study, Hung et al. found an opposite result, in that the overexpression of KIF14 impeded cell development and metastasis of NSCLC in vitro and vivo [49]. More research examining the cell biology of KIF14 in LUAD is clearly warranted. Large tumor suppressor kinase 2 (LATS2) promotes cell proliferation in NSCLC [50, 51]. Comparable findings were reported in other research that illustrated that as an independent prognostic biomarker of LATS2 in NSCLC, survival was significantly improved in the high expression cohort [52]. In addition, LATS2 interacted with YAP1 and restricted nuclear translocation of YAP1, which enhances transcription activity of PD-L1 and leads to immune escape in ovarian cancer [53]. Protein kinase C (PRKCE) has been found to be involved in metastasis and malignant transformation and is upregulated in several cancers, such as lung, breast, and gall bladder cancers [54–56]. PRKCE was also found to be associated with radiation sensitivity in LUAD [57]. However, limited research has been carried out regarding the role of TM6SF1 in tumorigenesis and cancer progression, and comprehensive examinations of its biological functions in lung cancer are necessary.

Although the above studies indicate that our GPS can be a valuable prognostic predictor for LUAD, the study encountered some limitations. While data accumulated from high-throughput analyses with a large sample size was applied optimally, confirmation via prospective studies is warranted. Additionally, the interactions between the four genes and GPX4, as well as the precise biological functions of these genes in ferroptosis should be explored experimentally.

## 5. Conclusions

In conclusion, a robust GPS was developed and validated in two independent datasets. Also, we built a prognostic nomogram by integrating the GPS and TNM stage, and it exhibited exceptional performance in forecasting the survival in patients with LUAD. The GPS was significantly related to multiple TICs and immune-related processes and pathways in the TME of LUAD. These four GPX4-related genes could become a guaranteed prognostic biomarker and a possible therapeutic target for LUAD in the future.

## Data Availability

The raw data of this study are derived from the TCGA database and GEO data portal (accession number: GSE72094), which are publicly available databases.

## Abbreviations

LUAD: Lung adenocarcinoma
GPX4: Glutathione peroxidase 4
TCGA: The Cancer Genome Atlas
EGFR-TKIs: EGFR tyrosine kinase inhibitors
NSCLC: Non-small-cell lung cancer
OS: Overall survival
GPS: GPX4-related prognostic signature
TME: Tumor microenvironment
GEO: Gene Expression Omnibus
FDR: False discovery rate
WGCNA: Weighted gene coexpression network analysis
DEGs: Differentially expressed genes
ROC: Receiver operating feature curve
ESTIMATE: Estimation of STromal and Immune cells in MAlignant Tumour tissues using Expression data
ssGSEA: Single-sample gene set enrichment analysis
GSEA: Gene set enrichment analysis
GDSC: Genomics of Drug Sensitivity in Cancer
IC50: Half-maximal inhibitory concentration
TICs: Tumor-infiltrating immune cells
KIF14: Kinesin family member 14
LATS2: Large tumor suppressor kinase 2
PRKCE: Protein kinase C.

## Supplementary Materials

Supplementary Materials Figure S1: heat map of differentially expressed gene samples between lung adenocarcinoma and normal tissues. Figure S2: gene selection using the least absolute shrinkage and selection operator model. (a) 10-fold cross-validation for the coefficients of 7 GPX4-related DEGs in the LASSO model. (b) X-tile analysis of the 6 selected GPX4-related prognostic DEGs. Figure S3: validation of nomogram based on GSE72094 dataset. (A) The nomogram plot. (B) The nomogram calibration curves in the years 1, 3, and 5. Figure S4: connection network for four GPX4-related prognostic genes and GPX4. Table S1: the clinical peculiarity of patients from the TCGA and GSE72094 datasets. Table S2: the results of Spearman's correlation analysis. Table S3: the prognostic gene list related to OS of the GDEGs in the TCGA-LUAD dataset. Table S4: multivariable Cox model outcomes of genes in the GPS. Table S5: GSEA in the low-risk score cohort. Table S6: GSEA in the high-risk score cohort. Table S7: the four GPX4-related prognostic genes in the TCGA-LUAD dataset.

## References

[1] Bray F., Ferlay J., Soerjomataram I., Siegel R. L., Torre L. A., Jemal A. (2018). Global cancer statistics 2018: GLOBOCAN estimates of incidence and mortality worldwide for 36 cancers in 185 countries. *CA: a Cancer Journal for Clinicians*.

[2] Travis W. D. (2011). Pathology of lung cancer. *Clinics in Chest Medicine*.

[3] Kleczko E. K., Kwak J. W., Schenkand E. L., Nemenoff R. A. (2019). Targeting the complement pathway as a therapeutic strategy in lung Cancer. *Frontiers in Immunology*.

[4] Wu Y., Zhangand S., Gong X. (2020). The epigenetic regulators and metabolic changes in ferroptosis-associated cancer progression. *Molecular Cancer*.

[5] Friedmann Angeli J. P., Krysko D. V., Conrad M. (2019). Ferroptosis at the crossroads of cancer-acquired drug resistance and immune evasion. *Nature Reviews. Cancer*.

[6] Seiler A., Schneider M., Förster H. (2008). Glutathione peroxidase 4 senses and translates oxidative stress into 12/15-lipoxygenase dependent- and AIF-mediated cell death. *Cell Metabolism*.

[7] Seibt T. M., Pronethand B., Conrad M. (2019). Role of GPX4 in ferroptosis and its pharmacological implication. *Free Radical Biology & Medicine*.

[8] Yang W. S., SriRamaratnam R., Welsch M. E. (2014). Regulation of ferroptotic cancer cell death by GPX4. *Cell*.

[9] Zhang X., Sui S., Wang L. (2020). Inhibition of tumor propellant glutathione peroxidase 4 induces ferroptosis in cancer cells and enhances anticancer effect of cisplatin. *Journal of Cellular Physiology*.

[10] Weijiao Y., Fuchun L., Mengjie C. (2021). Immune infiltration and a ferroptosis-associated gene signature for predicting the prognosis of patients with endometrial cancer. *Aging (Albany, NY.)*.

[11] Bersuker K., Hendricks J. M., Li Z. (2019). The CoQ oxidoreductase FSP1 acts parallel to GPX4 to inhibit ferroptosis. *Nature*.

[12] Ma C. S., Lv Q. M., Zhang K. R. (2021). NRF2-GPX4/SOD2 axis imparts resistance to EGFR-tyrosine kinase inhibitors in non-small-cell lung cancer cells. *Acta Pharmacologica Sinica*.

[13] Ni J., Chen K., Zhangand J., Zhang X. (2021). Inhibition of GPX4 or mTOR overcomes resistance to lapatinib via promoting ferroptosis in NSCLC cells. *Biochemical and Biophysical Research Communications*.

[14] Xu C., Sun S., Johnson T. (2021). The glutathione peroxidase Gpx4 prevents lipid peroxidation and ferroptosis to sustain Treg cell activation and suppression of antitumor immunity. *Cell Reports*.

[15] Drijvers J. M., Gillis J. E., Muijlwijk T. (2021). Pharmacologic screening identifies metabolic vulnerabilities of CD8+T cells. *Cancer Immunology Research*.

[16] Freire Boullosa L., van Loenhout J., Flieswasser T. (2021). Auranofin reveals therapeutic anticancer potential by triggering distinct molecular cell death mechanisms and innate immunity in mutant p53 non-small cell lung cancer. *Redox Biology*.

[17] Schabath M. B., Welsh E. A., Fulp W. J. (2016). Differential association of *STK11* and *TP53* with *KRAS* mutation- associated gene expression, proliferation and immune surveillance in lung adenocarcinoma. *Oncogene*.

[18] Langfelderand P., Horvath S. (2008). WGCNA: an R package for weighted correlation network analysis. *BMC Bioinformatics*.

[19] Li C. Y., Cai J., Tsaiand J. J. P., Wang C. C. N. (2020). Identification of hub genes associated with development of head and neck squamous cell carcinoma by integrated bioinformatics analysis. *Frontiers in Oncology*.

[20] Wang C. C. N., Li C. Y., Cai J. H. (2019). Identification of prognostic candidate genes in breast cancer by integrated bioinformatic analysis. *Journal of Clinical Medicine*.

[21] Huo J., Wuand L., Zang Y. (2021). Development and validation of a CTNNB1-associated metabolic prognostic model for hepatocellular carcinoma. *Journal of Cellular and Molecular Medicine*.

[22] Liu F., Liaoand Z., Song J. (2020). Genome-wide screening diagnostic biomarkers and the construction of prognostic model of hepatocellular carcinoma. *Journal of Cellular Biochemistry*.

[23] Yoshihara K., Shahmoradgoli M., Martínez E. (2013). Inferring tumour purity and stromal and immune cell admixture from expression data. *Nature Communications*.

[24] Xu F., Shenand J., Xu S. (2021). Integrated bioinformatical analysis identifies GIMAP4 as an immune-related prognostic biomarker associated with remodeling in cervical cancer tumor microenvironment. *Frontiers in Cell and Developmental Biology*.

[25] Liu J., Ma H., Meng L. (2021). Construction and external validation of a ferroptosis-related gene signature of predictive value for the overall survival in bladder cancer. *Frontiers in Molecular Biosciences*.

[26] Rooney M. S., Shukla S. A., Wu C. J., Getzand G., Hacohen N. (2015). Molecular and genetic properties of tumors associated with local immune cytolytic activity. *Cell*.

[27] Reimand J., Isserlinand R., Voisin V. (2019). Pathway enrichment analysis and visualization of omics data using g:Profiler, GSEA, Cytoscape and EnrichmentMap. *Nature Protocols*.

[28] Liu J., Wuand Z., Wang Y. (2020). A prognostic signature based on immune-related genes for cervical squamous cell carcinoma and endocervical adenocarcinoma. *International Immunopharmacology*.

[29] Shaurova T., Zhang L., Goodrichand D. W., Hershberger P. A. (2020). Understanding lineage plasticity as a path to targeted therapy failure in EGFR-mutant non-small cell lung cancer. *Frontiers in Genetics*.

[30] Liu Z., Wang L., Liu L. (2021). The identification and validation of two heterogenous subtypes and a risk signature based on ferroptosis in hepatocellular carcinoma. *Frontiers in Oncology*.

[31] Wang Z., Diao J., Zhao X., Xuand Z., Zhang X. (2021). Clinical and functional significance of a novel ferroptosis-related prognosis signature in lung adenocarcinoma. *Clinical and Translational Medicine*.

[32] Tang Y., Li C., Zhangand Y., Wu Z. (2021). Ferroptosis-related long non-coding RNA signature predicts the prognosis of head and neck squamous cell carcinoma. *International Journal of Biological Sciences*.

[33] Zhu L., Yangand F., Wang L. (2021). Identification the ferroptosis-related gene signature in patients with esophageal adenocarcinoma. *Cancer Cell International*.

[34] Can T. (2014). Introduction to bioinformatics. *Methods in Molecular Biology*.

[35] Zhou J., Guo H., Liu L. (2021). Construction of co-expression modules related to survival by WGCNA and identification of potential prognostic biomarkers in glioblastoma. *Journal of Cellular and Molecular Medicine*.

[36] Zhang Y., Zhang L., Xu Y., Wu X., Zhou Y., Mo J. (2020). Immune-related long noncoding RNA signature for predicting survival and immune checkpoint blockade in hepatocellular carcinoma. *Journal of Cellular Physiology*.

[37] Zhao Y., du T., du L. (2019). Long noncoding RNA LINC02418 regulates MELK expression by acting as a ceRNA and may serve as a diagnostic marker for colorectal cancer. *Cell Death & Disease*.

[38] Wu J., Li L., Zhang H. (2021). A risk model developed based on tumor microenvironment predicts overall survival and associates with tumor immunity of patients with lung adenocarcinoma. *Oncogene*.

[39] Zhang Z., Baoand S., Yan C., Hou P., Zhou M., Sun J. (2021). Computational principles and practice for decoding immune contexture in the tumor microenvironment. *Briefings in Bioinformatics*.

[40] Lei X., Lei Y., Li J. K. (2020). Immune cells within the tumor microenvironment: biological functions and roles in cancer immunotherapy. *Cancer Letters*.

[41] Chi A., Heand X., Hou L. (2021). Classification of non-small cell lung cancer’s tumor immune micro-environment and strategies to augment its response to immune checkpoint blockade. *Cancers*.

[42] Pinto R., Daniela P., Rosanna L. (2019). KRAS-driven lung adenocarcinoma and B cell infiltration: novel insights for immunotherapy. *Cancers*.

[43] Liu X., Wuand S., Yang Y., Zhao M., Zhu G., Hou Z. (2017). The prognostic landscape of tumor-infiltrating immune cell and immunomodulators in lung cancer. *Biomedicine & Pharmacotherapy*.

[44] Dai X., Luand L., Deng S. (2020). USP7 targeting modulates anti-tumor immune response by reprogramming tumor-associated macrophages in lung cancer. *Theranostics*.

[45] Zhernov I., Diez S., Braunand M., Lansky Z. (2020). Intrinsically disordered domain of kinesin-3 Kif14 enables unique functional diversity. *Current Biology*.

[46] Zhu Q., Renand H., Li X. (2020). Silencing *KIF14* reverses acquired resistance to sorafenib in hepatocellular carcinoma. *Aging (Albany NY)*.

[47] Singel S. M., Cornelius C., Zaganjor E. (2014). KIF14 promotes AKT phosphorylation and contributes to chemoresistance in triple-negative breast cancer. *Neoplasia*.

[48] Corson T. W., Zhu C. Q., Lau S. K., Shepherd F. A., Tsao M. S., Gallie B. L. (2007). KIF14 messenger RNA expression is independently prognostic for outcome in lung cancer. *Clinical Cancer Research*.

[49] Hung P. F., Hong T. M., Hsu Y. C. (2013). The motor protein KIF14 inhibits tumor growth and cancer metastasis in lung adenocarcinoma. *PLoS One*.

[50] Gu C., Chenand J., Dang X. (2021). Hippo pathway core genes based prognostic signature and immune infiltration patterns in lung squamous cell carcinoma. *Frontiers in Oncology*.

[51] Li W., Sunand M., Zang C. (2016). Upregulated long non-coding RNA AGAP2-AS1 represses LATS2 and KLF2 expression through interacting with EZH2 and LSD1 in non-small-cell lung cancer cells. *Cell Death & Disease*.

[52] Wu A., Liand J., Wu K. (2016). LATS2 as a poor prognostic marker regulates non-small cell lung cancer invasion by modulating MMPs expression. *Biomedicine & Pharmacotherapy*.

[53] Feng S., Sunand H., Zhu W. (2021). miR-92 overexpression suppresses immune cell function in ovarian cancer via LATS2/YAP1/PD-L1 pathway. *Clinical and Translational Oncology*.

[54] Zhang G., Wu J., Wang H., Jiangand W., Qiu L. (2020). Overexpression of microRNA-205-5p exerts suppressive effects on stem cell drug resistance in gallbladder cancer by down-regulating PRKCE. *Bioscience Reports*.

[55] Yang D., Heand Y., Wu B. (2020). Predictions of the dysregulated competing endogenous RNA signature involved in the progression of human lung adenocarcinoma. *Cancer Biomarkers*.

[56] Wang H., Gutierrez-Uzquizaand A., Garg R. (2014). Transcriptional regulation of oncogenic protein kinase C*ϵ* (PKC*ϵ*) by STAT1 and Sp1 proteins. *The Journal of Biological Chemistry*.

[57] Pu X., Wang L., Chang J. Y. (2014). Inflammation-related genetic variants predict toxicity following definitive radiotherapy for lung cancer. *Clinical Pharmacology and Therapeutics*.

