# Supplementary material for: Identification and Validation of a GPX4-Related Immune Prognostic Signature for Lung Adenocarcinoma
Source: J Oncol. 2022 May 17;2022:9054983. doi: 10.1155/2022/9054983 (PMC9130018; doi:10.1155/2022/9054983)
Supplement: Supplementary Materials — Figure S1: heat map of differentially expressed gene samples between lung adenocarcinoma and normal tissues. Figure S2: gene selection using the least absolute shrinkage and selection operator model. (a) 10-fold cross-validation for the coefficients of 7 GPX4-related DEGs in the LASSO model. (b) X-tile analysis of the 6 selected GPX4-related prognostic DEGs. Figure S3: validation of nomogram based on GSE72094 dataset. (A) The nomogram plot. (B) The nomogram calibration curves in the years 1, 3, and 5. Figure S4: connection network for four GPX4-related prognostic genes and GPX4. Table S1: the clinical peculiarity of patients from the TCGA and GSE72094 datasets. Table S2: the results of Spearman's correlation analysis. Table S3: the prognostic gene list related to OS of the GDEGs in the TCGA-LUAD dataset. Table S4: multivariable Cox model outcomes of genes in the GPS. Table S5: GSEA in the low-risk score cohort. Table S6: GSEA in the high-risk score cohort. Table S7: the four GPX4-related prognostic genes in the TCGA-LUAD dataset. [file 9054983.f1.zip › 9054983.f1/Table S1 (1).pdf]

Table S1: The clinical peculiarity of LUAD patients from the TCGA and GSE72094 dataset

Dataset	TCGA	GSE72094
Cohort	Training cohort	Validation cohort
Total patients	477	386
Age at diagnosis (y)	66(33-88)	66(38-89)
Gender		
Male/Female	220/257	168/218
Smoking		
Never/Ever	-	95/291
TNM stage		
I/II/III/IV/unknow	253/113/78/25/8	246/65/56/14/5
T stage		
T1/T2/T3/T4/TX	159/254/43/18/3	-
N stage		
N0/N1/N2/N3/NX	307/90/67/2/11	-
M stage		
M0/M1/MX	313/24/140	-
Survival status		
Dead/Alive	157/320	109/277
