# Supplementary material for: Identification and Validation of a GPX4-Related Immune Prognostic Signature for Lung Adenocarcinoma
Source: J Oncol. 2022 May 17;2022:9054983. doi: 10.1155/2022/9054983 (PMC9130018; doi:10.1155/2022/9054983)
Supplement: Supplementary Materials — Figure S1: heat map of differentially expressed gene samples between lung adenocarcinoma and normal tissues. Figure S2: gene selection using the least absolute shrinkage and selection operator model. (a) 10-fold cross-validation for the coefficients of 7 GPX4-related DEGs in the LASSO model. (b) X-tile analysis of the 6 selected GPX4-related prognostic DEGs. Figure S3: validation of nomogram based on GSE72094 dataset. (A) The nomogram plot. (B) The nomogram calibration curves in the years 1, 3, and 5. Figure S4: connection network for four GPX4-related prognostic genes and GPX4. Table S1: the clinical peculiarity of patients from the TCGA and GSE72094 datasets. Table S2: the results of Spearman's correlation analysis. Table S3: the prognostic gene list related to OS of the GDEGs in the TCGA-LUAD dataset. Table S4: multivariable Cox model outcomes of genes in the GPS. Table S5: GSEA in the low-risk score cohort. Table S6: GSEA in the high-risk score cohort. Table S7: the four GPX4-related prognostic genes in the TCGA-LUAD dataset. [file 9054983.f1.zip › 9054983.f1/Table S2 (1).pdf]

Table S2: The results of spearman's correlation analysis

Gene	cor	P-value	Gene	cor	P-value
A2M	-0.214	5.81E-07	H2AFZP3	-0.243	1.33E-08
ABCA8	-0.219	3.19E-07	H2AFZP4	-0.240	1.85E-08
ABCA9	-0.301	1.05E-12	HAGHL	0.330	4.88E-15
ABHD11	0.294	4.02E-12	HASPIN	-0.244	1.14E-08
ABI3BP	-0.231	6.77E-08	HCN2	0.258	1.42E-09
AC002128	-0.213	6.37E-07	HDHD3	0.257	1.58E-09
AC002128	-0.260	1.05E-09	HEATR1	-0.254	2.45E-09
AC003991	-0.245	8.83E-09	HECW2	-0.219	3.32E-07
AC004223	-0.236	3.24E-08	HEG1	-0.362	4.93E-18
AC004461	-0.249	5.08E-09	HELLS	-0.265	4.56E-10
AC004466	-0.219	3.05E-07	HIST2H2B	-0.222	2.04E-07
AC004832	-0.253	2.8E-09	HM13-IT1	-0.203	2.14E-06
AC004832	-0.256	1.93E-09	HMBS	0.303	7.58E-13
AC004837	-0.254	2.56E-09	HMGA1P2	-0.216	4.78E-07
AC004884	-0.223	1.96E-07	HMGB3P4	-0.254	2.42E-09
AC004890	-0.244	1.01E-08	HNRNPA3	-0.286	1.46E-11
AC004908	-0.236	3.41E-08	HOMER3	0.268	2.88E-10
AC005034	-0.236	3.51E-08	HOOK1	-0.253	3.08E-09
AC005062	-0.203	2.28E-06	HRASLS	0.212	7.32E-07
AC005154	-0.204	1.88E-06	HSP90B1	-0.268	2.76E-10
AC005256	0.240	1.9E-08	HSPA8P15	-0.254	2.47E-09
AC005326	-0.204	1.85E-06	HSPD1P11	-0.209	1.11E-06
AC005519	-0.262	7.83E-10	HSPE1P3	-0.205	1.82E-06
AC005522	-0.214	5.87E-07	HYAL2	0.316	7.02E-14
AC005730	-0.220	2.9E-07	IFNG-AS1	-0.233	4.77E-08
AC005828	-0.222	2.21E-07	IGF2BP3	-0.202	2.39E-06
AC005831	-0.257	1.67E-09	IGHJ3P	-0.204	1.89E-06
AC005839	-0.259	1.2E-09	IGHV3-79	-0.241	1.77E-08
AC005856	-0.240	1.85E-08	IGKJ5	-0.228	1E-07
AC006023	-0.225	1.43E-07	IKZF2	-0.288	1.08E-11
AC006111	-0.212	7.76E-07	IL18R1	-0.246	8.12E-09
AC006330	-0.241	1.63E-08	IL37	0.201	2.75E-06
AC006441	-0.272	1.71E-10	IL7R	-0.247	7E-09
AC006480	-0.239	2.16E-08	ILF2	0.208	1.2E-06
AC006504	-0.212	7.08E-07	IMMP1LP1	-0.243	1.24E-08
AC007000	-0.239	2.08E-08	INPP4B	-0.288	1.18E-11
AC007014	-0.203	2.08E-06	INSYN2	-0.217	4.11E-07
AC007038	-0.235	3.56E-08	IRF4	-0.215	5.06E-07
AC007191	-0.231	6.36E-08	IRF7	0.278	6.16E-11
AC007283	-0.208	1.22E-06	ISCA1P4	0.238	2.49E-08
AC007285	-0.245	9.1E-09	ISG15	0.253	3.01E-09
AC007365	-0.255	2.15E-09	ITFG1-AS1	-0.235	3.59E-08
AC007494	-0.246	8.29E-09	ITGA6-AS	-0.202	2.41E-06
AC007494	-0.234	4.19E-08	ITGA8	-0.205	1.67E-06
AC007546	-0.220	2.86E-07	ITPKA	0.218	3.69E-07
AC007569	-0.254	2.58E-09	ITPRID2	-0.298	1.98E-12
AC007639	-0.237	2.95E-08	ITPRIP	-0.206	1.59E-06
AC007731	-0.233	4.82E-08	JCAD	-0.201	2.92E-06
AC007906	-0.251	4.21E-09	JPT1	0.290	7.84E-12
AC007919	-0.224	1.65E-07	KCNAB1	-0.260	9.58E-10
AC007991	-0.226	1.33E-07	KCND2	-0.208	1.16E-06
AC007991	-0.208	1.15E-06	KCNQ1OT	-0.242	1.42E-08
AC008115	-0.243	1.26E-08	KCNT2	-0.204	1.99E-06
AC008243	-0.238	2.37E-08	KCTD12	-0.268	2.95E-10
AC008267	-0.265	4.44E-10	KDELR3	0.213	6.85E-07
AC008731	-0.283	2.81E-11	KIAA1211	-0.248	5.91E-09

AC008895	-0.242	1.52E-08	KIAA1324I	-0.248	6.52E-09
AC009005	0.288	1.15E-11	KIF14	-0.221	2.56E-07
AC009268	-0.220	2.93E-07	KIF20B	-0.248	5.71E-09
AC009704	-0.253	3.03E-09	KIF26B	-0.257	1.6E-09
AC009754	-0.241	1.64E-08	KISS1R	0.381	5.78E-20
AC009994	-0.232	5.57E-08	KLF13	-0.320	3.07E-14
AC010149	-0.252	3.57E-09	KLF4	-0.224	1.69E-07
AC010168	-0.264	5.58E-10	KLF9	-0.243	1.21E-08
AC010186	-0.223	1.87E-07	KNL1	-0.284	2.03E-11
AC010201	-0.238	2.57E-08	KNTC1	-0.278	5.85E-11
AC010210	-0.235	4.03E-08	KRTCAP2	0.341	5.38E-16
AC010320	-0.242	1.52E-08	KRTCAP3	0.276	8.62E-11
AC010378	-0.246	7.64E-09	LAGE3	0.276	8.26E-11
AC010422	-0.236	3.36E-08	LATS2	-0.290	7.83E-12
AC010524	-0.203	2.17E-06	LAX1	-0.255	2.28E-09
AC010834	-0.267	3.53E-10	LCOR	-0.328	6.9E-15
AC010976	-0.265	4.96E-10	LDB2	-0.250	4.9E-09
AC011373	-0.209	1.09E-06	LIFR	-0.239	2.08E-08
AC011465	-0.267	3.3E-10	LINC0021(	-0.257	1.53E-09
AC011742	-0.247	7.17E-09	LINC0041;	-0.218	3.54E-07
AC011815	-0.213	6.3E-07	LINC0046	0.250	4.35E-09
AC011825	-0.246	8.28E-09	LINC0047;	-0.231	6.94E-08
AC011933	-0.211	8.93E-07	LINC0051;	-0.216	4.58E-07
AC011933	-0.214	6.21E-07	LINC0062	-0.206	1.53E-06
AC012170	-0.243	1.34E-08	LINC0063(	-0.256	1.83E-09
AC012213	-0.229	8.19E-08	LINC0129(	-0.204	1.91E-06
AC012254	-0.226	1.21E-07	LINC0135!	-0.252	3.44E-09
AC012447	-0.249	5.18E-09	LINC0142(	0.217	4.25E-07
AC012513	-0.346	1.9E-16	LINC0147;	-0.246	7.62E-09
AC012640	0.327	8.03E-15	LINC0151!	-0.205	1.74E-06
AC013394	-0.243	1.22E-08	LINC0183(	0.229	8.74E-08
AC015799	-0.249	5.37E-09	LINC0196;	-0.298	2.14E-12
AC015849	-0.256	1.81E-09	LINC0246	-0.208	1.24E-06
AC015914	-0.237	3.02E-08	LINC0256(	0.231	6.12E-08
AC015923	-0.228	9.95E-08	LPAR2	0.282	3.1E-11
AC015987	-0.262	7.31E-10	LRP2BP	-0.225	1.49E-07
AC016027	-0.238	2.53E-08	LRRC20	0.294	4E-12
AC016590	-0.247	7.19E-09	LRRC37A1	-0.257	1.61E-09
AC016737	-0.224	1.57E-07	LRRFIP1P1	-0.323	1.85E-14
AC017002	-0.220	2.61E-07	LRRN3	-0.257	1.55E-09
AC018410	-0.243	1.18E-08	LSAMP	-0.287	1.42E-11
AC018682	-0.217	4.28E-07	LST1	0.313	1.36E-13
AC018766	-0.287	1.22E-11	MACC1-A	-0.220	2.87E-07
AC019226	-0.231	6.78E-08	MAD2L2	0.339	7.54E-16
AC019226	-0.222	2.09E-07	MAFG-DT	0.307	3.95E-13
AC020612	-0.262	8.03E-10	MAGI2-AS	-0.250	4.67E-09
AC020612	-0.274	1.19E-10	MAL2-AS:	-0.247	6.81E-09
AC020661	-0.232	6.01E-08	MARCKSL	-0.207	1.41E-06
AC020765	0.207	1.44E-06	MATR3	-0.262	7.6E-10
AC020910	-0.221	2.46E-07	MBNL1-A	-0.243	1.24E-08
AC020913	-0.236	3.15E-08	MCC	-0.362	5.53E-18
AC020916	-0.203	2.14E-06	MCM3AP-	-0.246	8.6E-09
AC021087	-0.235	3.7E-08	MCM8	-0.242	1.36E-08
AC022150	-0.284	2.15E-11	MCRIP2	0.395	2.11E-21
AC022211	-0.202	2.48E-06	MCTS2P	-0.248	5.71E-09
AC022336	-0.251	4.19E-09	MDK	0.206	1.58E-06
AC022784	-0.295	3.28E-12	MESP1	0.314	1.14E-13
AC022960	-0.251	3.9E-09	MESP2	0.204	2.02E-06

AC022973	-0.262	8.02E-10	METTL26	0.503	1.08E-35
AC023051	-0.235	3.83E-08	MFSD13B	-0.251	3.75E-09
AC023271	-0.244	1.07E-08	MFSD9	-0.225	1.45E-07
AC023590	-0.244	1.16E-08	MGST1	0.265	4.57E-10
AC023818	-0.276	8.24E-11	MIF	0.389	7.98E-21
AC023825	-0.224	1.65E-07	MINCR	0.209	1.02E-06
AC023906	-0.232	5.93E-08	MIR181A1	-0.235	3.87E-08
AC024267	-0.260	9.57E-10	MIR30C2	-0.231	6.41E-08
AC024267	-0.264	5.42E-10	MIR3174	-0.221	2.51E-07
AC025031	-0.256	1.98E-09	MIR3192	-0.291	6.25E-12
AC025043	-0.262	7.2E-10	MIR320B2	-0.261	8.64E-10
AC025287	-0.257	1.69E-09	MIR3657	-0.266	4.14E-10
AC025917	-0.265	4.63E-10	MIR378J	-0.238	2.42E-08
AC026202	-0.236	3.3E-08	MIR3942	-0.233	4.78E-08
AC026356	-0.258	1.32E-09	MIR4653	0.230	7.55E-08
AC026368	-0.262	7.77E-10	MIR4668	-0.225	1.44E-07
AC027176	-0.261	8.56E-10	MIR4677	-0.223	1.78E-07
AC027277	-0.215	5.15E-07	MIR548AA	-0.215	5.03E-07
AC027514	-0.262	7.26E-10	MIR579	-0.213	6.69E-07
AC027544	-0.259	1.22E-09	MIR593	-0.222	2.17E-07
AC027544	-0.267	3.72E-10	MIR643	-0.232	5.7E-08
AC027763	-0.221	2.38E-07	MIR645	-0.227	1.17E-07
AC027796	-0.244	1.13E-08	MIR6740	-0.226	1.25E-07
AC027796	-0.232	5.96E-08	MITF	-0.285	1.97E-11
AC027801	-0.219	2.97E-07	MKI67	-0.202	2.42E-06
AC032044	-0.254	2.6E-09	MMS22L	-0.336	1.52E-15
AC035139	-0.209	1.14E-06	MNX1-AS	0.285	1.81E-11
AC037459	-0.291	7.01E-12	MPHOSPH	-0.329	6.15E-15
AC040169	-0.240	1.96E-08	MPP6	-0.266	3.87E-10
AC040904	-0.205	1.74E-06	MRGBP	0.215	5.35E-07
AC040934	-0.220	2.63E-07	MROH6	0.223	1.76E-07
AC063965	-0.241	1.6E-08	MRPL12	0.429	2.45E-25
AC067817	-0.261	8.59E-10	MRPL13	0.235	3.62E-08
AC067931	-0.210	9.65E-07	MRPL15	0.220	2.66E-07
AC067945	-0.222	2.14E-07	MRPL24	0.387	1.62E-20
AC068025	-0.254	2.36E-09	MRPL36	0.337	1.14E-15
AC069257	-0.220	2.74E-07	MRPS12	0.281	3.84E-11
AC073052	-0.255	2.19E-09	MRTO4	0.275	9.31E-11
AC073107	-0.226	1.35E-07	MSI2	-0.337	1.16E-15
AC073210	-0.210	9.03E-07	MSRB1	0.287	1.38E-11
AC073333	-0.258	1.48E-09	MSRB3	-0.238	2.67E-08
AC073367	-0.254	2.49E-09	MTFP1	0.309	2.59E-13
AC074138	-0.254	2.49E-09	MTX1	0.325	1.23E-14
AC078778	-0.258	1.39E-09	MUC21	0.258	1.31E-09
AC078852	-0.253	2.94E-09	MXRA5	-0.214	5.87E-07
AC078883	-0.261	8.85E-10	MYBPH	0.358	1.17E-17
AC079601	-0.249	5.55E-09	MYH10	-0.258	1.39E-09
AC079684	-0.245	9.17E-09	MYLK	-0.293	4.38E-12
AC079921	-0.231	6.8E-08	MYOCD	-0.206	1.6E-06
AC083798	-0.242	1.48E-08	MZT2A	0.360	8.76E-18
AC083837	-0.205	1.77E-06	NAA25	-0.330	5.09E-15
AC087276	-0.263	6.4E-10	NAALADL	-0.231	6.63E-08
AC087294	-0.231	6.32E-08	NABP2	0.220	2.83E-07
AC087392	-0.218	3.56E-07	NADK2-A	-0.257	1.54E-09
AC087683	-0.241	1.74E-08	NARF	0.221	2.5E-07
AC090114	-0.283	2.8E-11	NARF-IT1	-0.211	8.62E-07
AC090517	-0.212	7.66E-07	NAT9	0.302	1E-12
AC090579	-0.206	1.61E-06	NAV1	-0.315	9.07E-14

AC090695	-0.276	8.75E-11	NAV2-AS2	-0.233	5.03E-08
AC090739	-0.243	1.19E-08	NAXE	0.341	5.37E-16
AC091057	-0.240	1.84E-08	NCAPD3	-0.211	8.33E-07
AC091057	-0.219	3.07E-07	NCBP2-AS	-0.222	2.17E-07
AC091390	-0.244	1.09E-08	NCKAP5	-0.290	8.56E-12
AC092168	-0.215	5.24E-07	NDST1	-0.289	9.05E-12
AC092171	-0.252	3.29E-09	NDUFB4P(	-0.246	7.64E-09
AC092279	-0.219	3.01E-07	NDUFS6	0.352	5.07E-17
AC092338	-0.251	3.77E-09	NEAT1	-0.226	1.32E-07
AC092436	-0.233	5E-08	NEB	-0.223	1.8E-07
AC092574	-0.239	2.08E-08	NEBL	-0.200	3.05E-06
AC092755	-0.260	9.73E-10	NECAB1	-0.229	8.37E-08
AC092802	-0.273	1.39E-10	NEGR1	-0.232	5.54E-08
AC092828	-0.229	9.03E-08	NEXN	-0.260	9.98E-10
AC092839	-0.241	1.71E-08	NFASC	-0.224	1.67E-07
AC092964	-0.216	4.42E-07	NFYC-AS1	-0.227	1.14E-07
AC093110	-0.260	1.01E-09	NHS	-0.240	1.91E-08
AC093278	-0.224	1.63E-07	NHSL1	-0.241	1.68E-08
AC093484	-0.202	2.39E-06	NME1	0.397	1.09E-21
AC093690	-0.243	1.23E-08	NME1-NM	0.275	9.49E-11
AC093724	-0.255	2.18E-09	NME2	0.437	2.69E-26
AC093788	-0.231	6.53E-08	NME4	0.370	8.69E-19
AC096720	-0.253	3.05E-09	NOL3	0.210	1.01E-06
AC096921	-0.272	1.67E-10	NOP16	0.236	3.38E-08
AC097372	-0.221	2.39E-07	NPM1P26	-0.240	1.8E-08
AC097460	-0.241	1.73E-08	NPM1P29	-0.241	1.54E-08
AC097634	-0.226	1.34E-07	NPM3	0.324	1.46E-14
AC097641	-0.219	2.97E-07	NPR3	-0.222	2.17E-07
AC098591	-0.236	3.1E-08	NR2F6	0.263	6.57E-10
AC098869	-0.261	8.56E-10	NRBF2P5	-0.258	1.45E-09
AC098934	-0.205	1.69E-06	NSD2	-0.287	1.26E-11
AC099343	-0.244	1.04E-08	NSUN5	0.285	1.98E-11
AC099778	-0.258	1.3E-09	NUDT1	0.241	1.64E-08
AC099811	-0.247	7.04E-09	NUDT17	0.245	9.15E-09
AC099811	-0.241	1.56E-08	NUDT8	0.337	1.07E-15
AC100821	-0.250	4.79E-09	OCIAD2	0.203	2.17E-06
AC100821	-0.225	1.4E-07	OCLM	-0.244	1.1E-08
AC103739	-0.266	4.21E-10	ODCP	-0.248	6.3E-09
AC104115	-0.230	7.69E-08	OLFML1	-0.210	9.99E-07
AC104667	0.254	2.36E-09	OSTCP8	-0.244	1.12E-08
AC104695	-0.222	2.21E-07	OXLD1	0.447	1.36E-27
AC105235	-0.224	1.69E-07	P2RY1	-0.261	9.38E-10
AC105339	-0.248	6.28E-09	P4HB	0.202	2.41E-06
AC106037	-0.255	2.17E-09	PA2G4P6	-0.239	2.2E-08
AC108058	-0.222	2.1E-07	PABPC1P1	-0.236	3.12E-08
AC108471	-0.243	1.18E-08	PAFAH1B3	0.220	2.77E-07
AC108704	-0.276	8.82E-11	PAIP1P1	-0.256	1.86E-09
AC108727	-0.249	5.58E-09	PCDH17	-0.290	7.89E-12
AC108860	-0.237	2.98E-08	PCDH9	-0.228	9.71E-08
AC109322	0.205	1.84E-06	PCDHGB7	-0.277	7.29E-11
AC109460	-0.228	1E-07	PCDHGC3	-0.285	1.96E-11
AC109583	-0.246	8.68E-09	PCNX2	-0.315	9.04E-14
AC110285	0.240	1.9E-08	PCSK4	0.288	1.19E-11
AC111170	-0.257	1.56E-09	PDCD2L	0.230	6.99E-08
AC112694	-0.246	7.77E-09	PDCD6	0.217	3.82E-07
AC112722	-0.203	2.24E-06	PDE5A	-0.329	5.42E-15
AC113139	-0.224	1.71E-07	PDE8B	-0.301	1.08E-12
AC113191	-0.311	1.76E-13	PDK1	-0.262	7.04E-10

AC114402	-0.250	4.46E-09	PDZD11	0.314	1.06E-13
AC114402	-0.239	2.06E-08	PDZD2	-0.246	8.21E-09
AC116552	-0.213	6.45E-07	PEAK1	-0.427	4.4E-25
AC117500	-0.256	2E-09	PEAR1	-0.226	1.32E-07
AC117503	-0.269	2.63E-10	PERM1	0.260	1.02E-09
AC125807	-0.241	1.76E-08	PFDN2	0.274	1.19E-10
AC127024	-0.236	3.28E-08	PFDN6	0.311	1.92E-13
AC127024	-0.229	8.21E-08	PHACTR2	-0.360	7.37E-18
AC127024	-0.247	7.34E-09	PHBP12	-0.258	1.32E-09
AC127070	-0.268	3.02E-10	PHBP19	-0.249	5.25E-09
AC130650	-0.202	2.35E-06	PHLDA2	0.338	9.54E-16
AC131009	-0.272	1.52E-10	PHLDB2	-0.247	7.13E-09
AC131159	-0.251	3.8E-09	PIK3CD-A	0.269	2.57E-10
AC131212	-0.283	2.6E-11	PIP5K1B	-0.201	2.63E-06
AC131649	-0.245	9.31E-09	PIP5KL1	0.295	3.54E-12
AC131971	-0.242	1.54E-08	PITPNA-A	0.216	4.7E-07
AC132192	-0.259	1.13E-09	PLAGL1	-0.254	2.57E-09
AC132938	-0.202	2.48E-06	PLCE1	-0.283	2.43E-11
AC133473	-0.240	2.02E-08	PLEKHA6	-0.204	1.9E-06
AC134349	-0.242	1.34E-08	PLEKHA8	-0.269	2.58E-10
AC134407	-0.254	2.46E-09	PLEKHH2	-0.277	6.61E-11
AC134407	-0.233	5.08E-08	PLPP2	0.302	8.87E-13
AC134775	-0.280	4.41E-11	PLPP3	-0.256	1.98E-09
AC135279	-0.250	4.55E-09	PLSCR4	-0.333	2.82E-15
AC136475	-0.237	2.78E-08	PMM2	-0.212	7.39E-07
AC138393	-0.220	2.62E-07	POF1B	-0.220	2.77E-07
AC138393	-0.234	4.5E-08	POLQ	-0.238	2.48E-08
AC138409	-0.223	1.94E-07	POLR2H	0.318	4.65E-14
AC138409	-0.218	3.52E-07	POLR2KP1	-0.255	2.27E-09
AC138466	-0.205	1.7E-06	POMK	-0.277	7.34E-11
AC138696	0.219	3.22E-07	POP7	0.311	1.86E-13
AC138932	-0.268	2.84E-10	POU2AF1	-0.214	5.57E-07
AC139720	-0.238	2.41E-08	PPIAP40	-0.230	7.16E-08
AC139887	-0.242	1.4E-08	PPIAP42	-0.225	1.52E-07
AC139887	-0.306	4.51E-13	PPIAP51	-0.239	2.1E-08
AC139887	-0.239	2.05E-08	PPIAP91	-0.243	1.33E-08
AC141002	-0.218	3.6E-07	PPP1R14B	0.363	4.51E-18
AC145146	-0.202	2.54E-06	PPP1R14B	0.362	5.79E-18
AC145343	0.305	6.06E-13	PPP1R16A	0.231	6.82E-08
AC145676	-0.219	3.18E-07	PPP1R35	0.339	6.75E-16
AC211433	-0.253	3.16E-09	PREX2	-0.278	5.88E-11
AC211476	-0.232	5.56E-08	PRICKLE2	-0.294	4.02E-12
AC233300	-0.276	8.47E-11	PRKAA2	-0.257	1.68E-09
AC243654	-0.242	1.42E-08	PRKCA-AS	-0.225	1.51E-07
AC243732	-0.231	6.64E-08	PRKCE	-0.208	1.27E-06
AC244033	-0.224	1.54E-07	PRKCQ	-0.211	8.7E-07
AC244093	-0.239	2.23E-08	PRKDC	-0.298	2.01E-12
AC244093	-0.254	2.72E-09	PRKG1	-0.309	2.49E-13
AC245014	-0.234	4.29E-08	PRR36	0.212	7.09E-07
AC245100	-0.253	3.02E-09	PRR7	0.207	1.37E-06
AC245100	-0.224	1.65E-07	PRR7-AS1	-0.219	3E-07
AC245100	-0.206	1.6E-06	PSD3	-0.337	1.12E-15
AC245884	-0.225	1.4E-07	PSMB4	0.323	1.84E-14
AC245884	-0.259	1.21E-09	PSMD10P	-0.249	5.43E-09
AC253576	-0.223	1.92E-07	PSMG3	0.367	1.87E-18
ACACB	-0.271	1.72E-10	PTGFRN	-0.304	6.69E-13
ADAM12	-0.204	1.92E-06	PTPN21	-0.288	1.13E-11
ADAM15	0.313	1.37E-13	PTPRB	-0.308	3.39E-13

ADAM28	-0.268	3.09E-10	PTRH2	0.241	1.56E-08
ADAMTS1	-0.221	2.29E-07	PUS7L	-0.296	2.91E-12
ADAMTS1	-0.244	1.14E-08	PUSL1	0.386	1.93E-20
ADAMTS9	-0.224	1.74E-07	PYCR1	0.280	4.62E-11
ADAMTSL	-0.219	3.07E-07	PYCR3	0.294	3.69E-12
ADAMTSL	0.280	4.63E-11	QKI	-0.285	1.72E-11
ADARB1	-0.336	1.38E-15	RAB25	0.310	2.42E-13
ADGRL2	-0.242	1.35E-08	RAB5IF	0.223	1.91E-07
AF117829	-0.233	5.3E-08	RAC3	0.291	6.9E-12
AF131215	-0.267	3.35E-10	RALGPS2	-0.212	7.12E-07
AF230666	-0.255	2.21E-09	RASGEF1E	-0.241	1.67E-08
AFAP1L1	-0.308	3.51E-13	RASSF2	-0.275	1.01E-10
AGTPBP1	-0.267	3.59E-10	RBM12B-/	-0.298	2.11E-12
AHNAK	-0.310	2.21E-13	RBMS3	-0.207	1.45E-06
AK1	0.231	6.84E-08	RCC1	0.205	1.72E-06
AL008718.	-0.247	6.89E-09	RDH10-A:	-0.229	8.16E-08
AL022067.	-0.233	4.91E-08	RECK	-0.294	3.74E-12
AL022097.	-0.240	1.94E-08	REEP6	0.413	2.07E-23
AL022157.	-0.212	7.72E-07	RF00019	-0.260	1.07E-09
AL023807.	-0.208	1.2E-06	RF00139	-0.225	1.4E-07
AL031009.	-0.225	1.35E-07	RF00212	-0.251	4.07E-09
AL031768.	-0.226	1.28E-07	RF00275	-0.226	1.29E-07
AL034550.	-0.236	3.11E-08	RF00404	-0.236	3.38E-08
AL049539.	-0.238	2.68E-08	RF00406	-0.202	2.54E-06
AL049869.	-0.269	2.44E-10	RF00409	-0.245	9.81E-09
AL050341.	-0.230	7.13E-08	RF00411	-0.269	2.36E-10
AL080317.	-0.212	7.6E-07	RF00422	-0.263	6.98E-10
AL080317.	-0.245	9.41E-09	RF00569	-0.237	2.74E-08
AL096828.	0.217	3.91E-07	RF01293	-0.239	2.05E-08
AL109614.	-0.269	2.37E-10	RFWD3	-0.245	9.41E-09
AL109628.	-0.252	3.56E-09	RGS14	0.274	1.19E-10
AL109811.	0.213	6.48E-07	RHBDL1	0.255	2.19E-09
AL110115.	-0.232	5.34E-08	RHOD	0.305	5.37E-13
AL121601.	-0.247	7.51E-09	RHOJ	-0.241	1.76E-08
AL121652.	-0.230	7.51E-08	RN7SKP27	-0.249	5.12E-09
AL121845.	-0.210	9.04E-07	RN7SKP28	-0.253	2.73E-09
AL121992.	-0.241	1.55E-08	RN7SKP3(	-0.225	1.54E-07
AL132780.	-0.241	1.76E-08	RN7SL192	-0.232	5.38E-08
AL132780.	-0.255	2.26E-09	RN7SL239	-0.246	8.47E-09
AL133243.	-0.245	9.94E-09	RN7SL333	-0.264	5.78E-10
AL133243.	-0.256	1.85E-09	RN7SL566	-0.203	2.28E-06
AL136115.	-0.257	1.54E-09	RN7SL67P	-0.205	1.69E-06
AL136115.	-0.249	5.46E-09	RN7SL809	-0.228	9.37E-08
AL136164.	-0.209	1.1E-06	RN7SL812	-0.239	2.32E-08
AL136221.	-0.241	1.71E-08	RN7SL81P	-0.251	3.72E-09
AL138752.	-0.250	4.3E-09	RNA5SP1(	-0.238	2.44E-08
AL138831.	-0.209	1.04E-06	RNA5SP12	-0.253	3.12E-09
AL139128.	-0.235	3.96E-08	RNA5SP28	-0.226	1.32E-07
AL139132.	-0.235	3.93E-08	RNASEH2/	0.249	5.55E-09
AL139353.	-0.227	1.06E-07	RNF144B	-0.228	9.11E-08
AL158801.	-0.229	8.65E-08	RNU1-124	-0.253	2.82E-09
AL158825.	-0.217	3.8E-07	RNU1-134	-0.242	1.52E-08
AL162274.	-0.207	1.34E-06	RNU4-25F	-0.209	1.04E-06
AL162431.	-0.222	2.18E-07	RNU6-10(	-0.249	5.44E-09
AL162578.	-0.247	7.49E-09	RNU6-115	-0.246	8.49E-09
AL162724.	-0.241	1.62E-08	RNU6-118	-0.224	1.65E-07
AL162724.	-0.224	1.67E-07	RNU6-12(	-0.220	2.67E-07
AL353593.	-0.225	1.52E-07	RNU6-127	-0.231	6.55E-08

AL353662.	-0.223	1.76E-07	RNU6-130	-0.244	1.06E-08
AL353662.	-0.220	2.86E-07	RNU6-190	-0.222	2E-07
AL353748.	-0.258	1.34E-09	RNU6-216	-0.282	2.85E-11
AL353801.	-0.242	1.49E-08	RNU6-282	-0.247	7.15E-09
AL353804.	-0.213	6.36E-07	RNU6-315	-0.256	1.85E-09
AL353807.	-0.209	1.13E-06	RNU6-341	-0.249	5.18E-09
AL354726.	-0.264	5.82E-10	RNU6-387	-0.209	1.05E-06
AL355075.	-0.238	2.51E-08	RNU6-415	-0.227	1.04E-07
AL355353	0.245	9.58E-09	RNU6-431	-0.276	8.79E-11
AL355488.	-0.243	1.23E-08	RNU6-548	-0.244	1.02E-08
AL356055.	-0.220	2.7E-07	RNU6-652	-0.267	3.35E-10
AL359075.	-0.203	2.12E-06	RNU6-658	-0.247	7.22E-09
AL359740.	-0.202	2.44E-06	RNU6-757	-0.235	3.71E-08
AL359880.	-0.246	8.05E-09	RNU6-834	-0.263	6.96E-10
AL359922.	-0.256	1.82E-09	RNU6-853	-0.223	1.8E-07
AL360091.	-0.232	5.56E-08	RNU6-856	-0.274	1.2E-10
AL365277.	-0.252	3.59E-09	RNU6-875	-0.231	6.47E-08
AL365356.	-0.252	3.19E-09	RNU6-890	-0.254	2.63E-09
AL365436.	-0.221	2.44E-07	RNU6-930	-0.259	1.11E-09
AL390719.	0.280	4.26E-11	RNU6ATA	-0.229	8.15E-08
AL391069.	0.240	1.82E-08	RNU6ATA	-0.221	2.58E-07
AL391994.	-0.247	6.82E-09	RNU6V	-0.219	3.25E-07
AL445187.	-0.250	4.9E-09	RNU7-75P	-0.241	1.64E-08
AL445248.	-0.248	6.02E-09	RNVU1-1	-0.227	1.16E-07
AL445435.	-0.252	3.43E-09	RNY1P9	-0.243	1.19E-08
AL445471.	-0.207	1.32E-06	RPL12P11	-0.233	4.74E-08
AL450384.	-0.244	1.06E-08	RPL12P15	-0.206	1.58E-06
AL451060.	0.214	5.83E-07	RPL23AP1	-0.211	8.95E-07
AL512343.	-0.264	5.93E-10	RPL23AP5	-0.237	2.8E-08
AL512506.	-0.263	6.42E-10	RPL29P14	-0.215	5.29E-07
AL512656.	-0.284	2.16E-11	RPL31P58	-0.241	1.77E-08
AL513327.	-0.223	1.99E-07	RPL36AP1	-0.237	2.73E-08
AL513327.	-0.230	7.05E-08	RPL7AP2	-0.279	5.11E-11
AL513365.	-0.237	2.92E-08	RPL7P21	-0.243	1.3E-08
AL513365.	-0.251	4.05E-09	RPL7P57	-0.273	1.35E-10
AL513523.	-0.215	5.29E-07	RPS12P20	-0.241	1.76E-08
AL583832.	-0.254	2.56E-09	RPS15AP1	-0.261	8.32E-10
AL589765.	0.200	3.05E-06	RPS15AP3	-0.212	7.12E-07
AL590006.	-0.238	2.65E-08	RPS16P5	-0.220	2.67E-07
AL590723.	-0.241	1.65E-08	RPS29P16	-0.220	2.62E-07
AL590762.	-0.249	5.37E-09	RPS3AP38	-0.258	1.46E-09
AL592148.	-0.222	2.2E-07	RPUSD1	0.216	4.64E-07
AL604028.	-0.279	5.42E-11	RRAS	0.251	4E-09
AL606489.	-0.243	1.3E-08	RSF1-IT2	-0.237	3.01E-08
AL662844.	-0.215	5.04E-07	RTN4RL2	0.292	5.24E-12
AL669831.	-0.243	1.31E-08	RUNX1T1	-0.295	3.17E-12
AL669831.	-0.238	2.63E-08	RUNX2	-0.241	1.59E-08
AL671986.	-0.248	5.78E-09	RUSC1	0.215	5.46E-07
AL691432.	-0.238	2.68E-08	RWDD4P1	-0.229	8.94E-08
AL731563.	-0.212	7.56E-07	SAC3D1	0.361	7.24E-18
ALDH1L2	-0.286	1.49E-11	SAMD5	-0.234	4.52E-08
ALDOA	0.210	9.54E-07	SAP30L-A	-0.217	3.81E-07
ALG10	-0.256	1.82E-09	SASH1	-0.403	2.9E-22
ALG13-AS	-0.248	6.1E-09	SCARNA1	-0.208	1.25E-06
ALG3	0.265	4.83E-10	SCARNA8	-0.214	5.93E-07
ALMS1-IT	-0.202	2.51E-06	SCN7A	-0.238	2.46E-08
AMOTL1	-0.347	1.27E-16	SDHAF3	0.220	2.81E-07
ANGPTL1	-0.276	8.97E-11	SDHAP3	0.206	1.63E-06

ANGPTL7	-0.256	1.9E-09	SEC24D	-0.228	9.91E-08
ANKRD10	-0.295	3.13E-12	SEC62-AS	-0.272	1.47E-10
ANKRD36	-0.261	8.2E-10	SELENOI	-0.224	1.65E-07
ANKRD36	-0.252	3.34E-09	SELP	-0.204	2.01E-06
ANKRD36	-0.274	1.18E-10	SEMA3D	-0.206	1.64E-06
ANKRD61	-0.269	2.48E-10	SEMA6A	-0.218	3.51E-07
AP000560	-0.247	7.48E-09	SERINC1	-0.313	1.23E-13
AP000577	-0.238	2.41E-08	SERTAD1	0.327	7.96E-15
AP000692	-0.277	7.47E-11	SETP5	-0.232	6.03E-08
AP000753	-0.252	3.49E-09	SGK494	-0.204	1.97E-06
AP000786	-0.253	3.04E-09	SIRT7	0.207	1.34E-06
AP000866	-0.235	3.78E-08	SLC12A9-	0.241	1.64E-08
AP000873	-0.219	3.18E-07	SLC22A18	0.273	1.45E-10
AP001001	-0.265	4.85E-10	SLC25A10	0.299	1.54E-12
AP001021	-0.209	1.12E-06	SLC25A39	0.379	9.14E-20
AP001033	-0.232	5.85E-08	SLC35B2	0.255	2.33E-09
AP001189	-0.202	2.39E-06	SLC39A14	-0.274	1.16E-10
AP001429	-0.238	2.49E-08	SLC39A4	0.303	7.57E-13
AP001432	-0.227	1.08E-07	SLC4A7	-0.306	4.28E-13
AP001469	-0.237	2.87E-08	SLC50A1	0.433	7.71E-26
AP001528	-0.200	2.99E-06	SLC52A2	0.265	4.98E-10
AP001528	-0.217	4.11E-07	SLFNL1-A	-0.207	1.41E-06
AP001767	-0.265	4.74E-10	SLIT2	-0.302	1.04E-12
AP001893	-0.262	7.07E-10	SLIT3	-0.203	2.19E-06
AP002336	-0.242	1.53E-08	SMG1P3	-0.264	5.52E-10
AP002812	-0.237	2.74E-08	SMIM22	0.364	3.44E-18
AP002907	-0.246	8.74E-09	SMYD5	0.210	9.47E-07
AP003072	-0.262	7.54E-10	SNHG15	0.262	8.15E-10
AP003086	-0.229	8.49E-08	SNHG17	0.232	6.09E-08
AP003170	-0.255	2.15E-09	SNORA11	-0.211	8.58E-07
AP005131	-0.245	9.74E-09	SNORA11	-0.217	4.03E-07
AP005131	-0.250	4.39E-09	SNORA14	-0.209	1.07E-06
AP005136	-0.240	1.9E-08	SNORA22	-0.200	3.06E-06
AP005899	-0.250	4.58E-09	SNORA22	-0.230	7.77E-08
AP1S1	0.278	5.52E-11	SNORA22	-0.206	1.48E-06
AP4B1-AS	-0.202	2.53E-06	SNORA23	-0.211	8.04E-07
ARAP1-AS	-0.251	3.68E-09	SNORA26	-0.237	2.84E-08
ARGFXP2	-0.249	4.92E-09	SNORA28	-0.247	7.16E-09
ARHGAP1	-0.221	2.29E-07	SNORA2A	-0.243	1.33E-08
ARHGAP2	-0.250	4.85E-09	SNORA33	-0.201	2.77E-06
ARHGAP3	-0.218	3.71E-07	SNORA53	-0.236	3.2E-08
ARHGAP6	-0.226	1.24E-07	SNORA55	-0.266	4.01E-10
ARHGEF37	-0.243	1.3E-08	SNORA58	-0.245	1E-08
ARHGEF6	-0.264	5.34E-10	SNORA5A	-0.217	4.24E-07
ARL13A	-0.248	6.45E-09	SNORA60	-0.205	1.64E-06
ARL4AP4	-0.245	8.88E-09	SNORA65	-0.217	3.99E-07
ARPC3P1	-0.249	4.91E-09	SNORA66	-0.246	8.22E-09
ASH1L-IT1	-0.248	6.28E-09	SNORA74	-0.201	2.91E-06
ASIC3	0.217	3.86E-07	SNORA75	-0.233	5.33E-08
ASPM	-0.239	2.17E-08	SNORA80	-0.232	6.1E-08
ATAD5	-0.290	7.47E-12	SNORD10	-0.252	3.59E-09
ATP5PBP5	-0.212	7.26E-07	SNORD11	-0.234	4.57E-08
AVL9	-0.237	2.85E-08	SNORD11	-0.231	6.77E-08
B3GALNT2	-0.319	4.22E-14	SNORD12	-0.234	4.48E-08
B4GALT2	0.244	1.09E-08	SNORD12	-0.239	2.24E-08
B4GALT3	0.204	1.9E-06	SNORD12	-0.202	2.45E-06
BACE2	-0.235	4E-08	SNORD15	-0.216	4.49E-07
BARD1	-0.205	1.81E-06	SNORD15	-0.214	6.02E-07

BBIP1P1	-0.266	4.04E-10	SNORD19	-0.248	6.24E-09
BCAS2P2	-0.225	1.38E-07	SNORD53	-0.248	6.1E-09
BCL2L12	0.280	4.42E-11	SNORD59	-0.231	6.68E-08
BIRC6-AS2	-0.247	6.72E-09	SNORD6	-0.222	2.2E-07
BMPR2	-0.396	1.42E-21	SNORD63	-0.269	2.75E-10
BNIP3P10	-0.226	1.27E-07	SNORD67	-0.224	1.68E-07
BNIP3P24	-0.223	1.94E-07	SNORD69	-0.224	1.65E-07
BNIP3P27	-0.239	2.2E-08	SNORD72	-0.207	1.34E-06
BNIP3P40	-0.222	2.07E-07	SNORD73	-0.246	8.03E-09
BNIP3P42	-0.215	5.46E-07	SNORD9	-0.225	1.49E-07
BOLA1	0.370	7.9E-19	SNORD94	-0.212	7.82E-07
BOLA3	0.291	6.16E-12	SNRK	-0.413	2E-23
BRCA1	-0.219	3.16E-07	SNRNP25	0.426	5.24E-25
BRCA2	-0.312	1.6E-13	SNRPE	0.201	2.73E-06
BTF3P12	-0.239	2.06E-08	SNRPGP10	0.228	9.54E-08
C11orf65	-0.234	4.07E-08	SNX18P3	-0.233	5.09E-08
C14orf132	-0.212	7.17E-07	SPATA20	0.212	7.38E-07
C17orf58	0.215	5.24E-07	SPATC1L	0.207	1.35E-06
C1orf115	-0.209	1.1E-06	SPATS2	-0.218	3.77E-07
C1orf35	0.373	3.82E-19	SPDYA	-0.249	5.18E-09
C1orf53	0.308	3.41E-13	SPDYE1	-0.257	1.58E-09
C1QBPP2	-0.217	3.93E-07	SPDYE5	-0.247	6.64E-09
C20orf194	-0.343	3.26E-16	SPTBN1	-0.352	4.93E-17
C2orf27A	-0.264	5.54E-10	SRM	0.248	6.53E-09
C4orf48	0.365	2.74E-18	SSR4	0.236	3.28E-08
C5orf38	0.213	6.82E-07	ST7-OT4	-0.238	2.43E-08
C9orf41-A	-0.256	1.81E-09	STAP2	0.378	1.22E-19
CALCRL	-0.237	2.76E-08	STARD13	-0.344	2.42E-16
CAPN12	0.235	3.98E-08	STARD9	-0.229	8.23E-08
CASS4	-0.207	1.31E-06	STEAP2	-0.207	1.37E-06
CBX4	0.205	1.71E-06	SULF1	-0.226	1.33E-07
CBX8	0.305	5.44E-13	SUMO2P1	-0.236	3.11E-08
CCDC167	0.267	3.58E-10	SUMO4	-0.245	9.79E-09
CCDC18	-0.262	7.22E-10	SUPT16HF	-0.223	1.8E-07
CCDC24	0.209	1.05E-06	SUSD4	0.224	1.64E-07
CCDC7	-0.245	8.96E-09	SVEP1	-0.261	8.44E-10
CCND2	-0.207	1.33E-06	SYCE1L	0.211	8.54E-07
CD52	0.207	1.33E-06	SYNE4	0.270	2.29E-10
CD93	-0.286	1.62E-11	SYNJ2	-0.340	5.52E-16
CD99P1	-0.238	2.37E-08	SYNM	-0.244	1.09E-08
CDCA2	-0.206	1.51E-06	SYNPO2	-0.299	1.52E-12
CELF2	-0.235	3.93E-08	SYNPR-AS	0.240	1.79E-08
CENPE	-0.210	1.01E-06	SYT12	0.228	1.02E-07
CENPF	-0.222	2.04E-07	TACC1	-0.382	5.4E-20
CERS6	-0.304	7.08E-13	TAPT1-AS	-0.201	2.74E-06
CHKB-DT	0.205	1.67E-06	TAS2R15P	-0.254	2.71E-09
CLDN3	0.232	5.85E-08	TAS2R20	-0.232	5.63E-08
CLDN4	0.294	4.14E-12	TATDN2P2	-0.325	1.33E-14
CLPTM1L	0.238	2.48E-08	TBL1XR1-.	-0.245	8.92E-09
CLSPN	-0.222	2.27E-07	TBX3	-0.236	3.44E-08
COA4	0.341	4.73E-16	TBX5	-0.222	2.11E-07
COA6	0.231	6.75E-08	TBX5-AS1	-0.210	9.43E-07
COL5A2	-0.214	6.1E-07	TCEAL3-A	-0.227	1.17E-07
COL6A3	-0.239	2.04E-08	TDGF1P5	-0.242	1.41E-08
COL6A5	-0.209	1.1E-06	TDRKH-AS	0.323	2.02E-14
COL6A6	-0.275	9.31E-11	TENM4	-0.244	1.15E-08
COMTD1	0.299	1.51E-12	TET1	-0.303	8.32E-13
COX6B1P5	-0.259	1.21E-09	TFPI2	0.285	1.86E-11

COX7A2P1	-0.261	8.39E-10	TGFBR2	-0.260	1.07E-09
CPED1	-0.269	2.66E-10	TGFBR3	-0.257	1.62E-09
CR936218	-0.265	4.57E-10	TGFBR3L	0.353	3.89E-17
CRABP2	0.250	4.75E-09	TH2LCRR	-0.223	2E-07
CRACR2A	-0.307	3.85E-13	THRA1/BT	-0.219	3.14E-07
CRIM1	-0.321	2.76E-14	TIMM8AP	0.243	1.21E-08
CRYZP1	-0.262	8.03E-10	TK1	0.211	8.67E-07
CXorf36	-0.214	5.64E-07	TLCD1	0.355	2.38E-17
CYC1	0.280	4.49E-11	TLR4	-0.236	3.32E-08
CYTOR	0.216	4.47E-07	TM6SF1	-0.244	1.13E-08
DACH1	-0.225	1.46E-07	TMED3	0.250	4.84E-09
DANT2	-0.205	1.73E-06	TMEM177	0.316	7.07E-14
DAP3	0.260	1.04E-09	TMEM47	-0.277	6.97E-11
DCST1-AS	0.230	7.42E-08	TMEM52	0.202	2.5E-06
DDX39A	0.232	5.46E-08	TMEM75	-0.215	4.96E-07
DECR2	0.275	1.04E-10	TMEM99	0.249	5.13E-09
DEFB131B	-0.277	6.82E-11	TNFRSF18	0.221	2.4E-07
DGAT1	0.253	2.86E-09	TNFSF12	0.269	2.6E-10
DIRAS1	0.233	4.91E-08	TNS1	-0.227	1.18E-07
DIXDC1	-0.328	7.1E-15	TRAF4	0.330	4.38E-15
DLEU1	-0.208	1.23E-06	TRAJ1	-0.241	1.66E-08
DLEU2	-0.203	2.33E-06	TRIM47	0.223	1.95E-07
DLG5	-0.205	1.7E-06	TRPT1	0.311	2.02E-13
DLX3	0.214	5.55E-07	TSPAN18	-0.210	9.54E-07
DNAJC10	-0.348	1.23E-16	TSTA3	0.202	2.36E-06
DOCK11	-0.286	1.57E-11	TTC28	-0.334	1.97E-15
DOCK4	-0.291	6.79E-12	TTC6	-0.216	4.28E-07
DOP1B	-0.228	9.38E-08	TTF2	-0.259	1.18E-09
DPRXP4	-0.214	5.55E-07	TTLL7	-0.218	3.69E-07
DPY19L1P	-0.255	2.17E-09	TUBA4A	0.217	4.16E-07
DPY19L1P	-0.218	3.63E-07	TUBB8P1	-0.234	4.16E-08
DTX2	0.208	1.27E-06	TUBG1	0.200	3.07E-06
DTYMK	0.274	1.09E-10	TXNDC12	-0.253	2.93E-09
DUBR	-0.264	5.33E-10	TXNDC17	0.471	7.46E-31
DUS1L	0.315	8.91E-14	TXNP6	-0.224	1.68E-07
DUSP23	0.255	2.05E-09	TYROBP	0.267	3.5E-10
DYRK2	-0.215	5.43E-07	U62317.4	0.207	1.38E-06
ECE2	0.295	3.34E-12	UBASH3B	-0.216	4.63E-07
ECM2	-0.287	1.35E-11	UBE2L4	-0.229	8.06E-08
EEF2KMT	0.214	5.75E-07	UBE2R2-A	-0.248	5.8E-09
EFCAB13	-0.220	2.63E-07	UBFD1	-0.210	9.2E-07
EFNA3	0.263	6.64E-10	UCN	0.203	2.22E-06
EFNA4	0.339	6.93E-16	UQCC2	0.242	1.41E-08
EIPR1-IT1	-0.242	1.4E-08	UQCC3	0.456	7.02E-29
ELMO3	0.256	1.87E-09	URB1	-0.210	9.9E-07
EML1	-0.269	2.73E-10	USP3-AS1	-0.239	2.29E-08
ENO1	0.225	1.47E-07	UTAT33	-0.201	2.93E-06
ENPP2	-0.261	8.87E-10	UTRN	-0.352	4.3E-17
ENTPD7	-0.324	1.41E-14	VCAN	-0.241	1.56E-08
EPB41L2	-0.399	6.54E-22	VGLL3	-0.248	6.11E-09
EPHA10	0.238	2.42E-08	VN1R42P	-0.211	8.83E-07
EPHX3	0.236	3.13E-08	VPS72	0.250	4.66E-09
ERCC6L	-0.234	4.17E-08	VWF	-0.229	8.03E-08
ERG	-0.242	1.45E-08	WASHC5-	-0.250	4.91E-09
ERI3-IT1	-0.248	6.37E-09	WDR46	0.204	1.94E-06
ETS1	-0.320	3.24E-14	WFDC3	0.236	3.14E-08
EXOSC4	0.366	2.12E-18	WISP1	-0.256	1.83E-09
EXOSC5	0.333	2.42E-15	WWC2	-0.251	3.92E-09

F12	0.206	1.52E-06	XBP1P1	-0.241	1.68E-08
FAM198B-	-0.233	5.26E-08	YDJC	0.219	3.23E-07
FAM241B	0.350	7.44E-17	YPEL5P2	-0.255	2.05E-09
FAM49A	-0.230	7.14E-08	Z69666.1	-0.237	2.98E-08
FAM83B	-0.243	1.24E-08	Z82243.1	-0.275	9.51E-11
FANCI	-0.200	3.12E-06	Z98885.3	-0.251	3.96E-09
FAR2	-0.219	2.93E-07	Z99129.1	-0.274	1.14E-10
FAT1	-0.285	1.99E-11	Z99129.4	-0.245	9.71E-09
FBP1	0.211	8.01E-07	ZBED6	-0.293	5.13E-12
FBXL16	0.239	2.05E-08	ZBTB20-A	-0.251	3.96E-09
FBXL6	0.229	8.5E-08	ZBTB41	-0.225	1.49E-07
FBXO2	0.214	6.17E-07	ZC3HAV1I	-0.261	9.47E-10
FCRL5	-0.265	4.76E-10	ZEB1	-0.355	2.3E-17
FDX1P1	-0.241	1.64E-08	ZEB2	-0.313	1.2E-13
FERMT2	-0.259	1.13E-09	ZFYVE9	-0.323	1.96E-14
FERP1	-0.201	2.66E-06	ZGRF1	-0.279	4.69E-11
FGD6	-0.200	3.12E-06	ZKSCAN2-	-0.206	1.64E-06
FGF2	-0.272	1.68E-10	ZNF106	-0.413	2.07E-23
FLAD1	0.282	3.32E-11	ZNF107	-0.229	8.16E-08
FLI1	-0.255	2.08E-09	ZNF124	-0.215	5E-07
FMO2	-0.203	2.18E-06	ZNF146	-0.285	2E-11
FO680682	-0.218	3.33E-07	ZNF215	-0.242	1.51E-08
FOXD3-AS	0.245	8.76E-09	ZNF29P	-0.209	1.06E-06
FRAS1	-0.250	4.57E-09	ZNF346-IT	-0.256	1.79E-09
FRGCA	0.327	8.8E-15	ZNF367	-0.201	2.86E-06
FRK	-0.296	2.62E-12	ZNF451-A	-0.254	2.36E-09
FRMD3	-0.270	2.12E-10	ZNF587	-0.320	3.03E-14
FRMD4A	-0.318	4.56E-14	ZNF724	-0.225	1.52E-07
FRY	-0.302	9.37E-13	ZNF850	-0.243	1.28E-08
FZD3	-0.289	1.01E-11	ZNF92	-0.253	2.94E-09
GAB1	-0.300	1.35E-12	ZP3	0.214	5.72E-07
GALE	0.222	2.24E-07	ZWILCH	-0.226	1.2E-07
GALNT7	-0.270	2.03E-10			
GAMT	0.443	3.84E-27			
GAPDHP6	-0.236	3.19E-08			
GAS5	0.230	7.21E-08			
GATA6	-0.237	3.03E-08			
GCAT	0.360	8.64E-18			
GEN1	-0.321	2.69E-14			
GFOD1	-0.310	2.34E-13			
GFPT1	-0.208	1.19E-06			
GIMAP5	-0.271	1.77E-10			
GIMAP8	-0.237	2.83E-08			
GMFG	0.221	2.3E-07			
GMPPA	0.231	6.53E-08			
GNAQ	-0.342	4.09E-16			
GPR174	-0.269	2.71E-10			
GPR18	-0.240	1.92E-08			
GPR82	-0.227	1.1E-07			
GRK5	-0.262	7.47E-10			
GTF2IP9	-0.275	1.03E-10			
GUCY1A2	-0.300	1.36E-12			
