# Supplementary material for: Identification and Validation of a GPX4-Related Immune Prognostic Signature for Lung Adenocarcinoma
Source: J Oncol. 2022 May 17;2022:9054983. doi: 10.1155/2022/9054983 (PMC9130018; doi:10.1155/2022/9054983)
Supplement: Supplementary Materials — Figure S1: heat map of differentially expressed gene samples between lung adenocarcinoma and normal tissues. Figure S2: gene selection using the least absolute shrinkage and selection operator model. (a) 10-fold cross-validation for the coefficients of 7 GPX4-related DEGs in the LASSO model. (b) X-tile analysis of the 6 selected GPX4-related prognostic DEGs. Figure S3: validation of nomogram based on GSE72094 dataset. (A) The nomogram plot. (B) The nomogram calibration curves in the years 1, 3, and 5. Figure S4: connection network for four GPX4-related prognostic genes and GPX4. Table S1: the clinical peculiarity of patients from the TCGA and GSE72094 datasets. Table S2: the results of Spearman's correlation analysis. Table S3: the prognostic gene list related to OS of the GDEGs in the TCGA-LUAD dataset. Table S4: multivariable Cox model outcomes of genes in the GPS. Table S5: GSEA in the low-risk score cohort. Table S6: GSEA in the high-risk score cohort. Table S7: the four GPX4-related prognostic genes in the TCGA-LUAD dataset. [file 9054983.f1.zip › 9054983.f1/Table S3 (1).pdf]

Table S3: The prognostic gene list related to OS of the GDEGs in the TCGA-LUAD dataset

Gene	K-M P value	HR	HR.95L	HR.95H	Cox P value
AC005856	0.049	0.553	0.317	0.966	0.037
DOCK4	0.012	0.841	0.743	0.951	0.006
KIF14	<0.001	1.16	1.075	1.252	<0.001
LATS2	0.03	1.061	1.009	1.116	0.019
MZT2A	0.039	1.047	1.006	1.089	0.022
PRKCE	0.018	0.71	0.571	0.882	0.002
TM6SF1	0.047	0.774	0.645	0.93	0.006
