# Supplementary material for: Identification and Validation of a GPX4-Related Immune Prognostic Signature for Lung Adenocarcinoma
Source: J Oncol. 2022 May 17;2022:9054983. doi: 10.1155/2022/9054983 (PMC9130018; doi:10.1155/2022/9054983)
Supplement: Supplementary Materials — Figure S1: heat map of differentially expressed gene samples between lung adenocarcinoma and normal tissues. Figure S2: gene selection using the least absolute shrinkage and selection operator model. (a) 10-fold cross-validation for the coefficients of 7 GPX4-related DEGs in the LASSO model. (b) X-tile analysis of the 6 selected GPX4-related prognostic DEGs. Figure S3: validation of nomogram based on GSE72094 dataset. (A) The nomogram plot. (B) The nomogram calibration curves in the years 1, 3, and 5. Figure S4: connection network for four GPX4-related prognostic genes and GPX4. Table S1: the clinical peculiarity of patients from the TCGA and GSE72094 datasets. Table S2: the results of Spearman's correlation analysis. Table S3: the prognostic gene list related to OS of the GDEGs in the TCGA-LUAD dataset. Table S4: multivariable Cox model outcomes of genes in the GPS. Table S5: GSEA in the low-risk score cohort. Table S6: GSEA in the high-risk score cohort. Table S7: the four GPX4-related prognostic genes in the TCGA-LUAD dataset. [file 9054983.f1.zip › 9054983.f1/Table S4 (1).pdf]

Table S4: Multivariable Cox model outcomes of genes in the GPS

ID	coef	HR	HR.95L	HR.95H	p value
KIF14	0.104	1.109	1.013	1.215	0.025
LATS2	0.058	1.059	1.015	1.106	0.008
PRKCE	-0.268	0.765	0.612	0.955	0.018
TM6SF1	-0.204	0.815	0.680	0.977	0.027
