# Supplementary material for: Identification and Validation of a GPX4-Related Immune Prognostic Signature for Lung Adenocarcinoma
Source: J Oncol. 2022 May 17;2022:9054983. doi: 10.1155/2022/9054983 (PMC9130018; doi:10.1155/2022/9054983)
Supplement: Supplementary Materials — Figure S1: heat map of differentially expressed gene samples between lung adenocarcinoma and normal tissues. Figure S2: gene selection using the least absolute shrinkage and selection operator model. (a) 10-fold cross-validation for the coefficients of 7 GPX4-related DEGs in the LASSO model. (b) X-tile analysis of the 6 selected GPX4-related prognostic DEGs. Figure S3: validation of nomogram based on GSE72094 dataset. (A) The nomogram plot. (B) The nomogram calibration curves in the years 1, 3, and 5. Figure S4: connection network for four GPX4-related prognostic genes and GPX4. Table S1: the clinical peculiarity of patients from the TCGA and GSE72094 datasets. Table S2: the results of Spearman's correlation analysis. Table S3: the prognostic gene list related to OS of the GDEGs in the TCGA-LUAD dataset. Table S4: multivariable Cox model outcomes of genes in the GPS. Table S5: GSEA in the low-risk score cohort. Table S6: GSEA in the high-risk score cohort. Table S7: the four GPX4-related prognostic genes in the TCGA-LUAD dataset. [file 9054983.f1.zip › 9054983.f1/Table S5 (1).pdf]

Table S5: GSEA in the low-risk score cohort

NAME	SIZE	ES	NES	NOM p-va	FDR q-val
Cell adhesion molecules cams	131	-0.66	-2.259	< 0.001	< 0.001
Intestinal immune network for IgA production	46	-0.801	-2.135	< 0.001	0.002
JAK STAT signaling pathway	155	-0.529	-2.109	< 0.001	0.003
Cytokine cytokine receptor interaction	264	-0.558	-2.084	< 0.001	0.003
Chemokine signaling pathway	188	-0.549	-2.04	< 0.001	0.005
Toll like receptor signaling pathway	102	-0.548	-2.007	< 0.001	0.007
B cell receptor signaling pathway	75	-0.581	-1.947	0.004	0.011
Natural killer cell mediated cytotoxicity	132	-0.506	-1.821	0.008	0.028
VEGF signaling pathway	76	-0.455	-1.813	< 0.001	0.028
T cell receptor signaling pathway	108	-0.519	-1.821	0.010	0.029

RANK AT MAX
4745
4745
8398
6227
6712
6494
4855
6592
5541
6193
