# Supplementary material for: Identification and Validation of a GPX4-Related Immune Prognostic Signature for Lung Adenocarcinoma
Source: J Oncol. 2022 May 17;2022:9054983. doi: 10.1155/2022/9054983 (PMC9130018; doi:10.1155/2022/9054983)
Supplement: Supplementary Materials — Figure S1: heat map of differentially expressed gene samples between lung adenocarcinoma and normal tissues. Figure S2: gene selection using the least absolute shrinkage and selection operator model. (a) 10-fold cross-validation for the coefficients of 7 GPX4-related DEGs in the LASSO model. (b) X-tile analysis of the 6 selected GPX4-related prognostic DEGs. Figure S3: validation of nomogram based on GSE72094 dataset. (A) The nomogram plot. (B) The nomogram calibration curves in the years 1, 3, and 5. Figure S4: connection network for four GPX4-related prognostic genes and GPX4. Table S1: the clinical peculiarity of patients from the TCGA and GSE72094 datasets. Table S2: the results of Spearman's correlation analysis. Table S3: the prognostic gene list related to OS of the GDEGs in the TCGA-LUAD dataset. Table S4: multivariable Cox model outcomes of genes in the GPS. Table S5: GSEA in the low-risk score cohort. Table S6: GSEA in the high-risk score cohort. Table S7: the four GPX4-related prognostic genes in the TCGA-LUAD dataset. [file 9054983.f1.zip › 9054983.f1/Table S6 (1).pdf]

Table S6: GSEA in the high-risk score cohort

Name	SIZE	ES	NES	NOM p-val	FDR q-val	RANK AT MAX
Cell cycle	124	0.726	2.291	< 0.001	0.001	6262
RNA degradation	59	0.641	2.120	< 0.001	0.005	6462
Base excision repair	35	0.714	2.057	< 0.001	0.005	5618
DNA replication	36	0.861	2.069	< 0.001	0.006	2715
Nucleotide excision repair	44	0.66	2.009	0.002	0.007	9025
Homologous recombination	28	0.759	1.996	0.002	0.007	8306
Pentose phosphate pathway	27	0.655	1.923	< 0.001	0.010	4301
Mismatch repair	23	0.788	1.955	< 0.001	0.011	2312
RNA polymerase	28	0.624	1.820	0.010	0.022	7635
